# Food Security Determinants and Coping Strategies Among Rural Households in Ada'a District, Central Ethiopia

**DOI:** 10.1002/fsn3.71588

**Published:** 2026-03-09

**Authors:** Alem Shumiye, Degefa Tolossa, Solomon Tsehay

**Affiliations:** ^1^ Addis Ababa University College of Social Sciences, Arts and Humanities Addis Ababa Ethiopia

**Keywords:** Ada’a District, coping strategies, determinants, food security, rural households

## Abstract

Achieving food security continues to be a persistent challenge for rural communities, even in seemingly food‐secure areas such as Ada'a District, Central Ethiopia. Using a quantitative cross‐sectional household survey, this study examined key determinants of multidimensional household food security and the coping mechanisms employed during food shortages in Ada'a District. Data were collected from 424 households, and a composite food security index was adapted from the World Food Programme's Consolidated Approach for Reporting Indicators of Food Security (CARI). Ordered logistic regression was used to identify determinants of food security, whereas a zero‐inflated Poisson (ZIP) model was used to assess factors affecting coping frequency. Twelve out of seventeen predictors were statistically significant in the food security model. Using the CARI household food security variable (1 = food secure to 4 = severely food insecure), positive associations, indicating a movement toward a greater likelihood of moderate or severe food insecurity, were observed for households headed by single individuals, with a higher proportion of children under 14 years of age, experiencing seasonal labor migration, being located farther from the farm to the main road, and reporting rainfall variability or pest and disease infestations. In contrast, negative associations, indicating a greater likelihood of being food secure or marginally food secure, were observed for households with older heads, larger farmland holdings, participation in community‐based organizations, access to extension services, adoption of high‐yield varieties, and access to irrigation. In the ZIP model, rainfall variability, market distance, and market price shocks increased the frequency of coping. Conversely, extension access and remittance receipt reduced coping. The logit component showed that higher income and larger farm size increased the likelihood of households avoiding coping behaviors. The findings highlight the need for integrated interventions that provide climate‐smart agriculture support, improve rural market infrastructure, strengthen extension services and community organizations, and facilitate access to remittances and financial services to reduce food insecurity and reliance on negative coping strategies. This study advances food security measurement by quantitatively combining a multidimensional index with robust modeling of coping behaviors, providing nuanced insights for policy in rural Ethiopian contexts.

## Graphical abstract

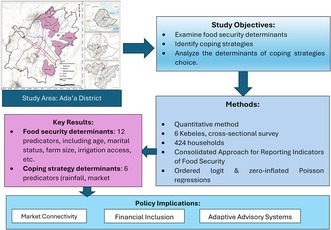

## 1 Introduction

Food security, defined as reliable access to sufficient, safe and culturally acceptable food (WFP 2009), is a basic human right and a key driver of sustainable development. Despite global commitments to achieve Zero Hunger by 2030, food insecurity, defined as the lack of reliable access to adequate food, remains a major global challenge, particularly in low‐ and middle‐income countries. In 2023, an estimated 713–757 million people globally, one in eleven, experienced hunger, with Africa disproportionately affected (FAO et al. 2024). Africa accounts for one in five hungry people worldwide, and projections suggest that 2.33 billion Africans (28.9% of the global population) faced moderate or severe food insecurity during that year (FAO et al. 2024). Although global food insecurity marginally declined from 28.4% in 2023 to 28.0% in 2024, Africa's situation deteriorated, with over 307 million people (20.2% of the population) experiencing hunger in 2024 (FAO et al. 2025). Ethiopia reflects this regional crisis. The country ranked 100th out of 113 nations in the 2022 Global Food Security Index (score: 0.49) and 102nd out of 127 countries in the 2024 Global Hunger Index (score: 26.2), indicating serious hunger levels (Economist Impact 2022; Welthungerhilfe and Concern Worldwide 2024). The 2023–2024 food crisis was driven by a convergence of localized conflict, macroeconomic instability, and climate extremes, resulting in uneven food access and forcing many households to rely on short‐term and erosive coping mechanisms (Global Network Against Food Crises 2024). These trends highlight that national‐level progress often conceals substantial household‐level vulnerabilities, underscoring the need for disaggregated, microlevel analysis. Despite progress over the past two decades, Ethiopia continues to face deep and spatially differentiated food security challenges that require targeted and evidence‐based interventions.

The food supply refers to the physical availability of food, whereas food security encompasses households' consistent physical, social, and economic access to adequate food. Nutritional security, in turn, concerns the quality and utilization of that food. In many rural areas, the food supply does not equate to nutritional security for all. Access constraints, vulnerability, and limited adaptive capacity, rather than food scarcity alone, often drive food insecurity, as emphasized by entitlement theory and livelihood‐based perspectives (Juliannisa et al. 2025; Ingram 2020; Maxwell and Smith 1992; Sen 1981; Sidhu et al. 2008). This conceptual distinction helps explain why food insecurity can persist even in areas with relatively high agricultural potential. Consequently, even agriculturally productive areas may experience significant household‐level food insecurity. This productivity–food security paradox is evident in Ada'a District of Central Ethiopia, a high‐potential farming area where aggregate production masks substantial intradistrict and household‐level disparities. Approximately 32.8% of farming households produce less than the minimum subsistence cereal requirements, whereas 43.4% cultivate less than one hectare, limiting their capacity to absorb production shocks (Ada'a District Agriculture Office 2024). The average cereal yield declined from 38.33 qt/ha in 2020 to 30.56 qt/ha in 2024, with Kebele^1^‐level yields ranging from 11.34 to 32.44 qt/ha, indicating marked spatial variation that intensifies production risk and shapes household vulnerability and coping behavior (Ada'a District Agriculture Office 2024). Pronounced rainfall variability, reaching coefficients of variation of 120% (1995), 167% (2018), and 77% (2024), further contributes to localized yield instability and heterogeneous levels of risk exposure (Ethiopian Meteorological Institute 2025). Empirical evidence also shows that food insecurity persists even in productive settings (Cahyono and Tokuda 2024; Melese et al. 2021; Nega et al. 2025; Rohne Till and Andersson 2025; Ruhyana et al. 2020; Sherka and Tsehay 2025), varying across socioeconomic groups and agroecological zones, with households often relying on coping strategies, underscoring the gap between aggregate output and actual food security.

Food security occurs when all people consistently have access to sufficient, safe, and nutritious food for an active and healthy life (FAO 2009; Peng and Berry 2019). Its four interconnected pillars, availability, access, utilization, and stability, must function simultaneously, although their relative importance varies across contexts. To operationalize these dimensions at the household level, researchers employ indicators such as the Food Consumption Score (FCS), Household Dietary Diversity Score (HDDS), Livelihood Coping Strategies (LCSs), Household Food Insecurity Access Scale (HFIAS), Food Insecurity Experience Scale (FIES), Months of Adequate Household Food Provisioning (MAHFP), Reduced Coping Strategy Index (rCSI), Household Hunger Scale (HHS), and Food Expenditure Share (FES), as well as the Household Food Balance Model (HFBM) or caloric adequacy, either individually or in combination. However, reliance on single indicators often captures only partial dimensions of food security. In some composite indices, strong performance in one dimension can mask weaknesses in another, a phenomenon described by Mutea et al. (2019) as the compensability effect. Conversely, studies using unaggregated indicators often fail to provide a unified food security status, limiting comprehensive vulnerability benchmarking. In this context, coping strategies provide critical insights into household responses to food stress and shocks, reflecting both short‐term survival mechanisms and longer‐term resilience capacity (Maxwell and Caldwell 2008; Mbhenyane et al. 2025; Sherka and Tsehay 2025).

To address these measurement limitations and examine drivers of multidimensional food security, this study adopts the consolidated approach for reporting indicators of food security (CARI), a globally recognized, harmonized, and field‐tested methodology developed by the World Food Programme (WFP 2015). The 2015 CARI framework integrates the FCS, FES, and LCSs into a single composite index that captures multiple food security dimensions within a four‐tier classification (see Section 3.3). This approach is particularly suitable for contexts such as Ada'a District, where apparent agricultural productivity coexists with household‐level vulnerability. To complement this structural assessment, the rCSI is applied to quantify short‐term coping mechanisms during food shortages, thereby strengthening the analysis of stability and vulnerability (Maxwell and Caldwell 2008). This combined approach enhances replicability, comparability, credibility, and analytical rigor while allowing for future validation and refinement of the composite indicator (Xu et al. 2025).

Previous research has examined the demographic, socioeconomic, institutional, infrastructural, and environmental determinants of household food security in Ethiopia (Alemayehu and Tesfaye 2024; Aweke et al. 2022; Beyene et al. 2024; Gebrehiwot et al. 2024; Sani and Kemaw 2019). While the literature provides valuable insights, empirical evidence remains highly context‐specific and methodologically fragmented, with many studies relying on single‐dimensional food security indicators that inadequately capture household vulnerability and coping behavior. This study addresses these gaps by applying a composite, multidimensional food security index and empirically examining the determinants of food security and coping strategies in Ada'a District, Central Ethiopia. By combining methodological rigor with contextual sensitivity, this study provides evidence to inform targeted and locally responsive interventions for rural households. Accordingly, this study is guided by the following central research question: How do demographic, socioeconomic, institutional, infrastructural, and environmental factors influence the food security status of rural households in Ada'a District, and what coping strategies do households employ during periods of food shortages? Specifically, it examines (i) key determinants of household food security; (ii) short‐term and long‐term coping strategies; and (iii) factors influencing the choice of coping strategies.

This study contributes to the literature in four key ways: contextually, it applies the standardized CARI at the micro level in Ada'a District, an area often perceived as food secure, in contrast with prior studies focused on more visibly food insecure or environmentally degraded areas; methodologically, it employs a quantitative, harmonized, and field‐tested CARI framework to capture availability, access, utilization, and stability within a single, interpretable four‐tier classification of food secure, marginally food secure, moderately food insecure, and severely food insecure; thematically, it links multidimensional food security measurement with household‐level determinants and coping strategies; and practically, it generates actionable, policy‐relevant evidence that can guide targeted food security and resilience interventions, especially in areas where vulnerability remains underrecognized.

The remainder of the study is structured as follows: Section 2 presents the literature review and analytical framework, Section 3 outlines the materials and methods, Section 4 describes the results and discussion, and Section 6 concludes the study with a summary of key findings and recommendations.

## 2 Literature Review

### 2.1 Theoretical Literature

The theoretical literature on food security has evolved from a supply‐centered perspective to a multidimensional, household‐focused understanding emphasizing access, utilization, stability, and resilience. Sen's (1981) Food Entitlement Decline (FED) theory highlights that food insecurity often arises from failures in access, via income, assets, or exchange, rather than aggregate shortages, and underscores how household decision‐making shapes coping strategies. This framework is particularly relevant in contexts such as Ada'a District, where food may be available but poverty limits access. In addition to FED, the sustainable livelihoods framework (SLF) links household assets and natural, financial, human, social, and physical, and vulnerability contexts shape both food security outcomes and coping capacity (Gichure et al. 2020; UNDP 2017).

Coping strategies reflect households' responses to entitlement failures and asset depletion, ranging from reversible adjustments to irreversible asset sales. Environmental–climate factors, such as rainfall variability and land degradation, undermine asset productivity and increase vulnerability, explaining why poorer households rely on more erosive strategies. Food systems and institutional factors shape the range of coping options available, with access to markets, infrastructure, and supportive policies enabling adaptive and reversible responses (Clapp et al. 2022). Resilience theory frames these dynamics by highlighting which strategies allow households to absorb shocks, adapt, and maintain long‐term food security. Together, these perspectives show that household food security and coping are determined by the interplay of asset endowment, environmental stressors, and systemic support, with stronger assets and institutional access promoting sustainable, adaptive strategies. The literature suggests that no single theoretical framework fully captures the multidimensional nature of household food security, which in this context is measured via CARI. Building on this, the study adopts the FED framework as its core lens, complemented by the SLF, environmental–climate, and food systems theories. Together, these perspectives provide an integrated approach to examine how household assets, environmental shocks, institutional support, and systemic food dynamics shape both food security outcomes and the coping strategies employed by households in Ada'a District.

### 2.2 Empirical Literature Review

The empirical literature on household food security in Ethiopia identifies multiple, often context‐specific determinants, with demographic factors showing inconsistent effects. For example, household head age has been associated with positive (Alemayehu and Tesfaye 2024; Aweke et al. 2022), insignificant (Cahyono and Tokuda 2024; Hailu 2023; Pakravan‐Charvadeh et al. 2022; Ruhyana et al. 2020), or negative effects (Sani and Kemaw 2019), reflecting a trade‐off between accumulated experience and declining labor capacity. Similarly, family size has mixed effects: larger households may face greater food insecurity (Cahyono and Tokuda 2024; Gebrehiwot et al. 2024; Hailu 2023; Ruhyana et al. 2020), likely because of greater consumption pressure; however, in some contexts, they benefit from pooled labor and income, reducing insecurity (Sani and Kemaw 2019), whereas others report no significant association (Beyene et al. 2024), suggesting that dependency and labor effects may offset each other depending on household composition and livelihood context.

Socioeconomic determinants of household food security exhibit mixed and context‐dependent effects. Factors such as landholding size, marital status, improved seed use, and migration experience are generally linked to enhanced food security (Beyene et al. 2024; Cahyono and Tokuda 2024; Gebrehiwot et al. 2024; Ruhyana et al. 2020), suggesting the role of productive assets and social capital in supporting household resilience. In contrast, farmland size, livestock ownership, and education have been associated with negative outcomes (Aweke et al. 2022; Gazuma and Astatike 2019), indicating that access to resources alone may be insufficient without favorable livelihood conditions and market integration. In some cases, education has no significant effect (Pakravan‐Charvadeh et al. 2022), suggesting that human capital alone may be insufficient. Taken together, evidence linking food insecurity to employment, assets, and farm inputs (Welderufael 2014), alongside findings emphasizing income diversification (Sani and Kemaw 2019), suggests that socioeconomic factors influence food security indirectly through household livelihood pathways and the institutional and market contexts in which they operate, rather than through uniform, direct effects.

Institutional and infrastructural factors influence household food security in conditional and pathway‐specific ways. While credit access and cooperative membership often improve food security (Beyene et al. 2024; Gebrehiwot et al. 2024), likely by supporting consumption smoothing and productive investment, other studies associate credit with increased food insecurity (Aweke et al. 2022; Gazuma and Astatike 2019), potentially due to indebtedness and weak targeting. Similarly, irrigation and market proximity enhance food security (Sani and Kemaw 2019) only where households can effectively utilize these services. Environmental shocks, particularly rainfall variability and pest incidence, directly undermine food availability and stability. Together, these findings underscore that institutional, infrastructural, and environmental factors shape food security differently. A brief overview of selected comparative empirical literature is provided in Annex A in Data S1. As summarized therein, a key limitation of prior studies is their reliance on single‐dimensional indicators or binary classifications of food security status (Alemayehu and Tesfaye 2024; Aweke et al. 2022; Cahyono and Tokuda 2024). Such approaches fail to capture the multidimensional nature of household food security and the gradations required for the effective targeting of interventions. Although some studies partially integrate multiple indicators (Beyene et al. 2024; Moreda et al. 2018), they tend to retain dimension‐specific analyses, which limits comparability across households and precludes a unified assessment of food security outcomes.

Recent studies have addressed these limitations by constructing composite food security indices via diverse methods, such as energy intake and share of income spent on food (Ruhyana et al. 2020), principal component analysis with min–max normalization (Guyalo et al. 2022), equal weighting of experience‐based questions from multiple indicators (Kassie et al. 2024), combining HFIAS and FCS (Kerbo et al. 2024), or aggregating standardized FCS and HDDS scores (Oburu et al. 2024). Other approaches include aggregating standardized HDDS, FCS, and MAHFP scores while subtracting food insecurity measures (Mutea et al. 2019) or emphasizing extreme deprivation by contrasting maximum and minimum scores (Sahu et al. 2017). Despite these advances, most of the literature has focused on demographic and socioeconomic factors, with limited attention given to infrastructural and environmental factors.

Existing composite measures are often ad hoc, with variability in indicator selection, weighting, and aggregation, which allows compensability that can mask severe deprivation in key food security pillars. Similarly, binary classifications fail to capture the depth of household vulnerability. Empirical evidence remains limited on how household characteristics, socioeconomic status, and shocks, including climate variability, market disruptions, and livelihood‐related shocks, influence the frequency, intensity, and determinants of consumption‐based coping strategies. This study addresses these gaps by adopting a transparent, four‐tiered CARI index that integrates access, availability, utilization, and stability while empirically examining demographic, socioeconomic, institutional, infrastructural, and environmental factors identified in the literature and relevant to the study area to assess their effects on household food security in Ada'a District. In addition, it fills the knowledge gap and contextual gap by examining the determinants of consumption‐based coping strategies.

### 2.3 Analytical Framework

**FIGURE 1:**
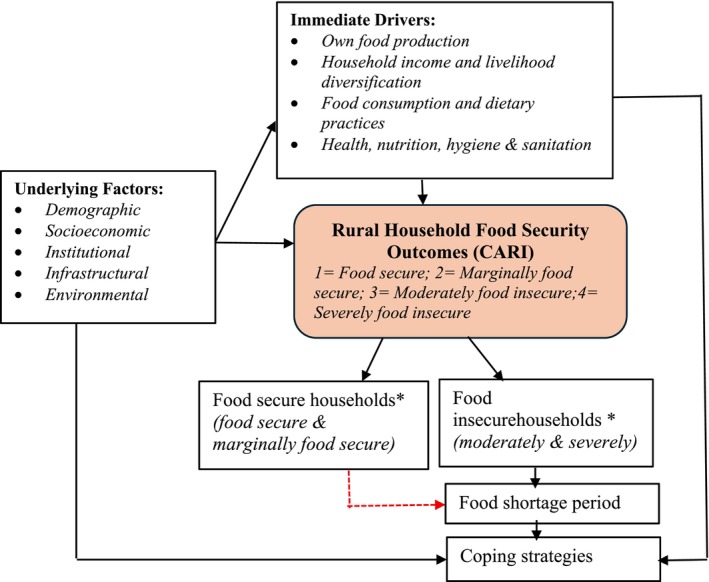
Analytical framework of the study. *The binary classification was adopted from Maxwell et al. (2013) and WFP (2015). *Source:* Author construction on the basis of a review of the literature.

The analytical framework (Figure 1) guiding this study draws on the reviewed theoretical and empirical literature. This study highlights key underlying factors that determine household food security and the coping strategies households adopt during food shortages, as well as the immediate drivers, such as their own food production and household income, that shape these strategies. In the framework, households were classified as food secure (food secure and marginally secure) or food insecure (moderate and severe) on the basis of Maxwell et al. (2013) and WFP (2015). The framework also captures short‐ and long‐term coping mechanisms adopted by both food secure and food insecure households. The red dotted line illustrates that even food households may face temporary food gaps and respond with coping mechanisms, reflecting the fluid and dynamic nature of food security. The framework also captures short‐ and long‐term coping mechanisms used by both food secure and food insecure households.

## 3 Materials and Methods

### 3.1 Study Area

**FIGURE 2:**
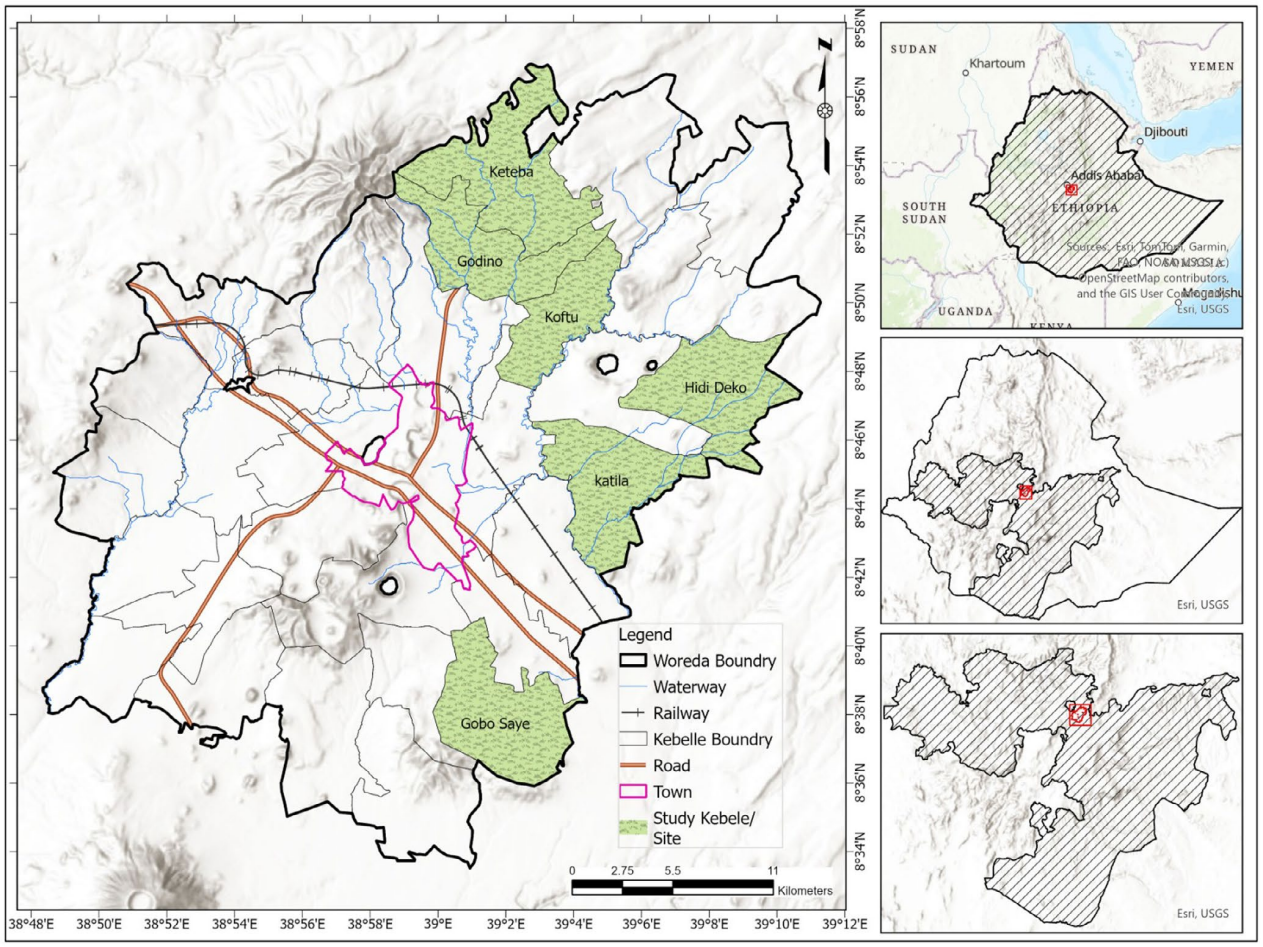
Map of Ada'a District showing study Kebeles. *Source:* Adapted from CSA (2007) and the Humanitarian Data Exchange (HDX) (2024).

Ada'a District (Figure 2) is located 45 km southeast of Addis Ababa in the East Shoa Zone, Oromia Region. The district spans an area of 708.49 km^2^, with elevations ranging from 1600 to over 3100 m above sea level (Ada'a District Finance Office 2023). Geographically, it lies between 8°34′ 0′′N and 8°58′ 0′′N latitude and 38°48′ 0′′E and 39°12′ 0′′E longitude, encompassing diverse landscapes and communities. According to the 2024 Central Statistical Services projection, the district has a population of 188,181, with 48% females (Ethiopian Statistical Services 2024). It consists of 24 rural and four urban *Kebeles* and is bordered by Akaki to the northwest, Gimbichu to the northeast, Lome to the east, and Dugda Bora to the south.

Ada'a District was purposively selected for this study because of its agricultural significance and the analytical leverage it provides. It has relatively high cereal production potential compared with neighboring districts, as evidenced by high teff ( *Eragrostis tef* ) and wheat ( *Triticum aestivum* ) yields reported by the Ada'a District Agriculture Office (2024). This context of high aggregate productivity, however, is marked by a critical productivity–food security paradox: Despite the district's output, 32.8% of households produce below the minimum subsistence requirement, and 43.4% cultivate less than one hectare (Ada'a District Agriculture Office 2024). This juxtaposition of high potential with prevalent vulnerability makes Ada'a ideal setting for examining the study's core objectives, namely, the drivers of multidimensional household food security (using CARI), the spectrum of associated short‐term and long‐term coping strategies, and hidden vulnerabilities not apparent from aggregate statistics. Consequently, the district provides a strategic case study to examine the gap between regional production and household food security and to generate evidence on the factors driving this disconnect and the coping strategies households adopt during food shortages. Data from the Ethiopian Meteorological Institute (2025) indicate that the average annual rainfall in Ada'a District is 713.84 mm, with two main rainy seasons: spring (April–May) and summer (June–August). Mixed farming, including crop production and livestock rearing, is the primary livelihood strategy, with subsistence farming as the main food source (Belay 2018).

### 3.2 Research Methods

#### 3.2.1 Research Design

This study employed a quantitative cross‐sectional research design, which is cost‐effective and allows the assessment of determinants of household food security and coping strategies at a single point in time. This design makes it suitable for capturing a snapshot of household conditions in Ada'a District, although it does not establish causality or capture changes over time.

#### 3.2.2 Sampling Strategy and Sample Size Determination

**FIGURE 3:**
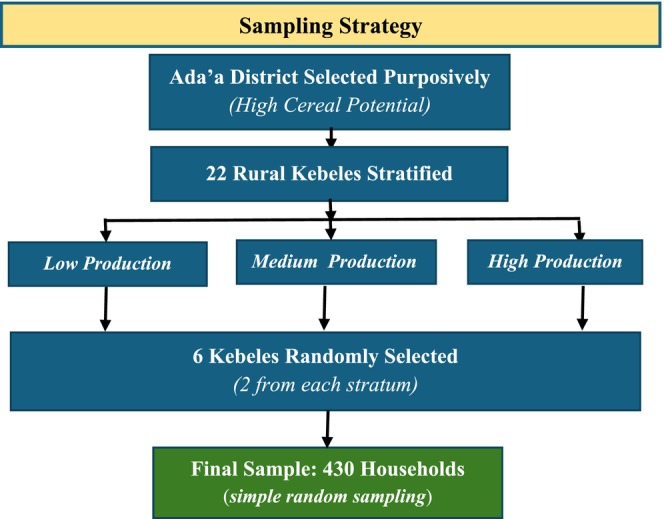
Flowchart of the sampling strategy. *Source:* Authors' construction.

A three‐stage sampling strategy integrating purposive, stratified, and random sampling methods was employed to ensure representativeness and analytical rigor (Figure 3). First, Ada'a District was purposively chosen for its presumed high cereal production potential, making it relevant for examining household food security and coping strategies. Second, the 22 rural Kebeles were first stratified into high, medium, and low cereal production categories on the basis of the Ada'a Agriculture Bureau classification. Six Kebeles were then randomly selected from these strata to ensure representation across production levels. Finally, within each selected Kebele, households engaged in mixed farming were selected via probability–proportional random sampling from household lists provided by the selected *Kebeles*. Households outside these *Kebeles* or not involved in farming were excluded.

The target population consisted of 5563 rural households, and the sample size was determined via Yamane's (1967) formula with a 5% margin of error, resulting in 373 households. To account for potential nonresponses and missing data, the sample size was increased by 15%, resulting in a final sample of 430 households, which were allocated proportionally to the size of each Kebele, as presented in Table 1.

$$n=\frac{N}{1+Ne^{2}}=\frac{5,563}{1+5,563{\left(0.05\right)}^{2}}=373$$

**TABLE 1:** Sample size determination.

Agricultural potential/stratum	Rural *Kebele*	Total household size	Percentage	Household size/Kebele
Low	Gobesay	810	15	63
	Deko	815	15	63
Medium	Godino	1013	18	78
	Koftu	567	10	44
High	Keteba	1126	20	87
	Katila	1232	22	95
	Total	5563	100	430

*Source:* Author construction (2024) on the basis of data from Ada'a District Agriculture Office.

#### 3.2.3 Data Collection and Quality Assurance

Primary quantitative data were collected between April and May 2024 through face–to‐face structured interviews conducted by trained enumerators under supervision. The questionnaire covered demographic, socioeconomic, institutional, infrastructural, and environmental factors. It also included questions on food security and coping strategies adapted from WFP (2015) to ensure consistency and comparability. The questionnaire was pretested (*n* = 30) to ensure reliability and validity, with refinements made for clarity and interview duration. Secondary data on food production, market prices, rainfall, and relevant statistics from published studies were obtained from reputable sources to complement and validate the primary data. District‐level monthly rainfall data (1995–2024) from the Ethiopian Meteorological Institute were cross‐checked with nearby stations and historical averages, and missing or outlier values were verified. Market prices were collected from multiple sources per Kebele, standardized, cross‐checked, and supplemented with reference averages. Recall bias was minimized by using clearly defined recall periods, standardized prompts, and triangulation with secondary data, although some bias may remain. Social desirability bias was mitigated by ensuring that respondents were confident in confidentiality and framing questions neutrally, whereas interviewer bias was minimized through thorough training and consistent questionnaire administration.

#### 3.2.4 Ethical Considerations

Ethical approval for the study was obtained from the Institutional Review Board of the College of Development Studies, Addis Ababa University (Approval No. 064/03/2023; March 8, 2024). Prior to data collection, informed consent was secured from all participants, and confidentiality, anonymity, voluntary participation, and the right to withdraw were fully ensured.

#### 3.2.5 Data Analysis

Descriptive statistics were used to summarize predictor and outcome variables, with means, standard deviations, and coefficients of variation reported for continuous variables and frequencies, percentages, and graphical presentations for categorical variables. The standardized anomaly index (SAI), which measures rainfall deviations relative to the long‐term average and captures both the direction and intensity of anomalies, was calculated as the difference between annual rainfall and the long‐term mean divided by the standard deviation. Positive values indicate above‐average rainfall, whereas negative values indicate below‐average rainfall. To characterize rainfall variability further, the coefficient of variation (CV) was computed as the ratio of the standard deviation to the mean, providing a normalized measure of interannual rainfall variability. Together, these indices allow the assessment of how both rainfall irregularities and variability are related to household food security. Household food security indicators and coping strategies were analyzed descriptively following established calculation criteria. Mean differences in continuous predictors across the four food security categories were examined via one‐way analysis of variance (ANOVA), followed by Tukey's HSD post hoc tests, whereas chi‐square tests were used to assess associations between categorical predictors and food security status.

The multivariate relationship between predictor variables and ordinal food security status was analyzed via an ordered logistic regression model. Model diagnostics included tests of the proportional odds assumption, overall model fit, and multicollinearity, assessed via Spearman's pairwise correlations and variance inflation factors (VIFs). Determinants of coping frequency were analyzed via a zero‐inflated Poisson (ZIP) model, which was selected after alternative count‐data specifications, including standard Poisson, negative binomial, and zero‐inflated negative binomial models, were compared on the basis of the Akaike information criterion (AIC) and Bayesian information criteria (BIC), with the model exhibiting the lowest values chosen. All analyses were conducted via Stata version 17 and SPSS version 27, with statistical significance evaluated at the 1%, 5%, and 10% levels.

### 3.3 Household Food Security Index (FSI) and Coping Strategies

This study used a composite measure based on the CARI of the World Food Program (WFP 2015). To capture the complementary dimensions of household coping, both livelihood coping strategies ‐food security (LCS‐FS) and the reduced coping strategies index (rCSI) were employed. The LCS‐FS reflects longer‐term coping capacity and economic vulnerability through stress, crisis, and emergency strategies affecting future productivity, whereas the rCSI captures short‐term, acute coping behaviors during immediate food shortages, weighted by severity. Using both indices provides a comprehensive assessment of household coping, capturing chronic vulnerabilities and acute responses without redundancy. The computation of these indicators is described below.

- Food consumption score (FCS): The FCS, adapted from WFP (2008), is used to assess household food access. Eight food groups, cereals, pulses and nuts, vegetables, fruits, meat and fish, milk and milk products, sugar, and oil, are considered, along with their diversity, frequency, and nutritional significance. A 7‐day recall period was used to gather household data, which measured how frequently each food group was consumed over the previous week. Each food group's frequency is multiplied by its nutritional weight, which ranges from 0.5 (fats, sugar, and oil) to 4 (meat, fish, and milk products), to determine the FCS. On the basis of their FCS values, households were divided into three groups: “acceptable” (> 35), “borderline” (21.5–35), and “poor” (≤ 21) (WFP 2008).
- Food expenditure share (FES): The FES, adapted from WFP (2015), measures the proportion of total monthly household expenditure on food and serves as a proxy for economic vulnerability. It included both purchased and unpurchased food, such as own‐production, in‐kind assistance, or gifts, valued at local market prices, or, if unavailable, at average prices in reference to *Kebele*. Household expenditures were collected for the past 30 days (for food and short‐term nonfood items) and the past 6 months (for long‐term nonfood items, data were averaged over six months). The monthly food expenditure (MFE) and two nonfood expenditure variables (NFME1 for the short term and NFME2 for the long term) were aggregated, and the FES was calculated as the MFE divided by the sum of the MFE, NFME1, and NFME2. On the basis of WFP (2015) cutoffs, households were classified as food secure (FES < 50%), marginally food secure (50%–65%), moderately food insecure (65%–75%), or severely food insecure (≥ 75%).
- Livelihood coping strategies—food security (LCS‐FS): The LCS‐FS measures the proportion of households that used livelihood coping strategies during the past 30 days or had exhausted such strategies within the preceding 12 months (WFP 2015). It is derived from household responses to experiences of livelihood stress and asset depletion over the specified recall periods. Both the recent use of coping strategies and the exhaustion of strategies in the past year indicate a reduced capacity to cope with future shocks. The ten coping strategies were grouped into three severity levels: stress, crisis, and emergency. Stress strategies (e.g., borrowing money or selling nonproductive assets) indicate emerging vulnerability and reduced resilience due to resource depletion. Crisis strategies (e.g., consuming seed stocks or reducing agricultural inputs) undermine future productivity and human capital. Emergency strategies (e.g., selling land or begging) have severe and often irreversible consequences for livelihoods. Households that reported no use of coping strategies were classified as food secure. The respondents were also asked to explain their reasons for not adopting specific strategies. In line with WFP (2015) guidance, strategies used previously but discontinued within the past 12 months were also considered adopted.

Following Food Security Cluster (2021) guidelines, households were categorized on the basis of the most severe strategy employed; for example, a household using both stress and crisis strategies was classified under crisis. On the basis of coping responses and the WFP (2015) CARI framework, households were classified into four LCS‐FS categories: score 1 (food secure; no coping strategies), score 2 (marginal food security; stress strategies), score 3 (moderate food insecurity; crisis strategies), and score 4 (severe food insecurity; emergency strategies).

d Reduced copings strategies index (rCSI): The rCSI is calculated by summing the frequency of five specific coping behaviors that households use when facing short‐term food shortages, each weighted by its universal severity. These behaviors and their severity weights typically include eating less‐preferred foods (1), borrowing food or money (2), limiting portion sizes at meals (1), reducing adult consumption so that children can eat (3), and reducing the number of meals per day (1) (Maxwell and Caldwell 2008). The rCSI score is calculated by multiplying the frequency of each coping strategy by its severity weight and summing the results. While no universal thresholds exist, higher scores indicate more severe coping. This study follows Maxwell et al. (2013), classifying rCSI scores as low (0–2), medium (3–12), or high (13+).

**FIGURE 4:**
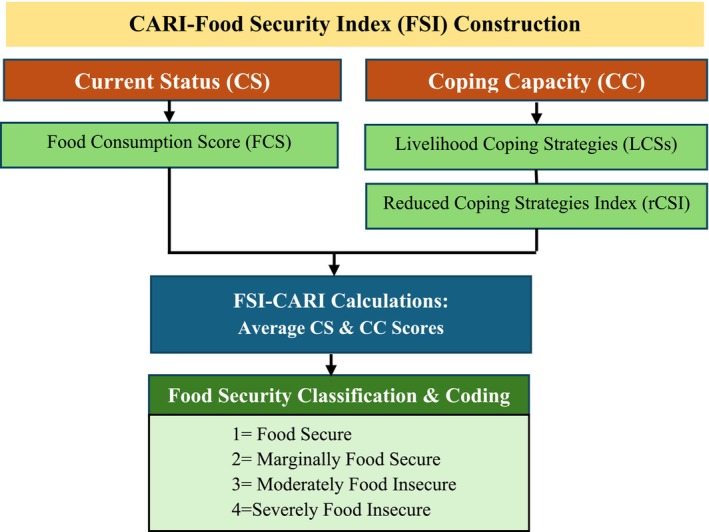
CARI‐FSI construction. *Source:* Authors' construction.

The CARI methodology constructs a multidimensional food security index (FSI) by combining household‐level indicators from the current status (CS) and coping capacity (CC) domains (WFP 2015), as illustrated in Figure 4.

The CC score was based on the FCS, while the CC score was calculated as the average of the LCS‐FS and FES scores. The FSI was then computed as the average of the CS and CC scores, rounded to the nearest whole number. Households were classified according to their FSI as food secure (0–1.25), marginally food secure (1.5–2.25), moderately food insecure (2.5–3.25), or severely food insecure (3.5–4.0). Accordingly, household food security status was measured via the CARI classification and coded as an ordinal variable: 1 = food secure, 2 = marginally food secure, 3 = moderately food insecure, and 4 = severely food insecure, with higher categories (3 and 4) indicating greater levels of food insecurity. For simplified analysis and comparison, households were also classified into dichotomous variables: “food secure” (food secure and marginally secure) or “food insecure” (moderate and severe) (WFP 2015; Maxwell et al. 2013), acknowledging the limitations of this aggregation.

### 3.4 Econometric Model Specification

#### 3.4.1 Analysis of Factors Influencing Household Food Security

An ordered logistic regression model was used to assess the effects of multiple predictors on the likelihood that households would fall into each food security category.

Let $\gamma_{\mathrm{jk}}$ denote the probability of $j^{\mathrm{th}}$ households falling into the $k^{\mathrm{th}}$ food security category. We model the log‐odds of being in category k or lower relative to being in higher categories. For each k, the log‐odds $\gamma_{\mathrm{jk}}$ is calculated relative to the higher categories. Mathematically, the log‐odds is modeled as a linear function of the predicator variables x, which is simplified as follows:

(1) $$\gamma_{j1}=\ln\left(\frac{\gamma_{1}}{1-\gamma_{1}}\right)=\ln\left(\frac{\gamma_{1}}{\gamma_{2}+\gamma_{3}+\gamma_{4}}\right)=\alpha_{1}+\beta^{T}x=e^{\alpha_{1}+\beta^{T}x}$$

while $\beta^{T}x$ is a vector of covariates, the $\alpha_{1}$ intercept of the first category.

Similarly,

(2) $$\gamma_{j2}=\ln\left(\frac{\gamma_{1}+\gamma_{2}}{1-\gamma_{1}-\gamma_{2}}\right)=\ln\left(\frac{\gamma_{1}+\gamma_{2}}{\gamma_{3}+\gamma_{4}}\right)=\alpha_{2}+\beta^{T}x=e^{\alpha_{2}+\beta^{T}x}$$

(3) $$\gamma_{j3}=\ln\left(\frac{\gamma_{1}+\gamma_{2}+\gamma_{3}}{1-\gamma_{1}-\gamma_{2}-\gamma_{3}}\right)=\ln\left(\frac{\gamma_{1}+\gamma_{2}+\gamma_{3}}{\gamma_{4}}\right)=\alpha_{3}+\beta^{T}x=e^{\alpha_{3}+\beta^{T}x}$$

(4) $$\gamma_{j4}=\ln\left(\frac{\gamma_{1}+\gamma_{2}+\gamma_{3}+\gamma_{4}}{1-\gamma_{1}-\gamma_{2}-\gamma_{3}-\gamma_{4}}\right)=\ln\left(\gamma_{1}+\gamma_{2}+\gamma_{3}+\gamma_{4}\right)=\alpha_{4}+\beta^{T}x=e^{\alpha_{4}+\beta^{T}x}$$

For each threshold k, the general structure of the model can be summarized as follows:

$$\begin{array}{c} \gamma_{\mathrm{jk}}=\ln\left(\frac{\sum_{i=1}^{k}\gamma_{i}}{1-\sum_{i=1}^{k}\gamma_{i}}\right)=\alpha_{k}+\beta^{T}x=e^{\left(\alpha_{k}+\beta^{T}x\right)} \end{array}$$

where $\gamma_{\mathrm{jk}}$ is the cumulative probability of household j being in category k or lower (relative to higher‐to‐higher categories), $\alpha_{k}$ is the intercept for category k, $\beta^{T}x$ represents the linear combination of predictors, and $\sum_{i=1}^{k}\gamma_{i}$ represents the full probability mass across all categories. The outcome variable $Y_{j}$ (food security status for household j) is assumed to be related to predictor $x_{1},x_{2},x_{3},\ldots \ldots ,x_{17}$ through a linear relationship for each category k:

$$Y_{j}=\alpha_{k}+\beta_{1}x_{1}+\beta_{2}x_{2}+\ldots +\beta_{16}x_{17}+\varepsilon_{i}$$

where $\alpha_{k}$ is the intercept for category k; $\beta_{1}$ and $\beta_{2},\beta_{3},\ldots ,\beta_{17}$ are the regression coefficients associated with the predicators $x_{1}$ and $x_{2},x_{3},\ldots ,x_{17}$; and $\varepsilon_{i}$ is the error term for household j. Operational definitions and the hypothesized relationships between predictors and the outcome variable are presented in Annex B in Data S1. In the model, the dependent variable is coded 1 = food secure to 4 = severely food insecure. Accordingly, a negative coefficient indicates a movement toward a greater likelihood of being food secure or marginally food secure, whereas a positive coefficient indicates a greater likelihood of being moderately or severely food insecure.

#### 3.4.2 Determinants of Consumption‐Based Coping Frequency

Food shortages in rural areas are frequently caused by shocks that impact individual households (idiosyncratic) or entire communities (covariate). Depending on how severe and frequent the shocks are, households respond by implementing a variety of short‐ or long‐term coping strategies. This study examined the factors affecting short‐term consumption‐based coping mechanisms. We created an outcome variable on the basis of five typical consumption‐based coping behavior questions adapted from Maxwell and Caldwell (2008) to record household responses to temporary food shortages or financial stress. These questions assessed coping strategies used over the seven days preceding the survey. The consumption‐based coping frequency (CBCF), a count variable that ranges from 0 (no coping strategies used) to 5 (high coping frequency), was created from these values. A higher CBCF indicates greater vulnerability, whereas a lower frequency suggests stronger resilience.

Several count data models that are frequently used in the literature have been tested to investigate the factors that influence CBCF among rural households in Ada'a District: standard Poisson (SP), zero‐inflated Poisson (ZIP), negative binomial (NB), and zero‐inflated negative binomial (ZINB) models. After these alternatives were explored, the ZIP model was selected as the preferred specification on the basis of empirical evidence. According to the ZIP model, the observed outcome $Y_{i}$, which represents the CBCF for household i, is produced by combining two processes: a Poisson count process with mean $\lambda_{i}$ that determines the frequency of coping for the remaining households with probability $\left(1-\pi_{i}\right)$ and a binary process that determines whether the household belongs to a “certain‐zero” group (i.e., never uses coping strategies), with probability $\pi_{i}$. The probability distribution of the ZIP is as follows:

$$P\left(Y_{i}=0\right)=\pi_{i}+\left(1-\pi_{i}\right)e^{\lambda_{i}}$$

$$P\left(Y_{i}=y_{i}\right)=\left(1-\pi_{i}\right)+\frac{e^{\lambda_{i}}\lambda_{i}^{y_{i}}}{y_{i}},\text{for}\;y_{i}>0$$

where $\lambda_{i}=\exp\left(X_{i}^{T}\beta \right)$ is the expected count of coping strategies used by household i, $\pi_{i}=\frac{\exp\left(Z_{i}^{T}\gamma \right)}{1+\exp\left(Z_{i}\gamma \right)}$ is the probability that household i belongs to the structural zero group, and $X_{i}$ and $Z_{i}$ are vectors of household‐level predicators affecting the count and zero‐inflation parts of the model, respectively. γ is the vector of estimated coefficients that shows how each variable in $Z_{i}$ affects the likelihood of being a structural zero. Guided by the theoretical and empirical literature, we identified key predictors of coping frequency. The expected effects and underlying rationales are summarized in Annex C in Data S1. The incidence rate ratios (IRRs) from the count component of the ZIP model were used to interpret the effects of the predictors, indicating how a one‐unit change in a variable affects the expected count of the outcome. Specifically, an IRR > 1 indicates an increase, an IRR < 1 indicates a decrease, and an IRR = 1 indicates no effect after controlling for other variables.

## 4 Results and Discussion

We first provide an initial overview of the associations between predictors and food security status via descriptive statistics, which do not account for confounding factors. Both categorical and continuous predictors are analyzed, and food security indicators, including the CARI‐FSI, are examined via one‐way ANOVA for continuous variables and chi‐square tests for categorical variables. Differences across the four food security categories are reported, followed by a summary of food security indicators via the composite CARI‐FSI. Next, we present ordinal logistic regression results on the determinants of household food security, supported by model diagnostic tests to ensure the net effect of each predictor and the robustness of the model. Finally, we describe short‐ and long‐term coping strategies adopted during food shortages and report a zero‐inflated Poisson (ZIP) regression examining the determinants of consumption‐based coping frequencies.

### 4.1 Descriptive Analysis of Explanatory Variables

The study included 430 participants; after excluding six incomplete observations during data cleaning, the final sample consisted of 424 respondents (response rate: 98.6%). Table 2 presents one‐way ANOVA results examining the associations between continuous predictors and multidimensional food security status among households in Ada'a District (*n* = 424).

**TABLE 2:** One‐way ANOVA of continuous predictors and the multidimensional CARI, Ada'a District (*n* = 424).

Variables	FS mean (S.D.)	MFS mean (S.D.)	MFI mean (S.D.)	SFI mean (S.D.)	Overall mean (S.D.)	ANOVA result—*F* value
Age of household head (years)	50.07 (8.17)	47.65 (9.25)	45.22 (7.54)	44.90 (8.49)	47.64 (8.77)	6.19*
Highest educational status of household head (years)	6.07 (2.87)	6.39 (2.68)	4.92 (2.38)	5.53 (3.04)	5.96 (2.76)	5.82*
Proportion of children aged 14 years and under	0.48 (0.14)	0.47 (0.15)	0.65 (0.11)	0.67 (0.07)	0.52 (0.16)	49.79*
Farmland size (in hectares)	1.18 (0.90)	1.07 (0.78)	0.88 (0.47)	0.79 (0.47)	1.05 (0.76)	3.90*
Number of crops grown	2.88 (1.37)	3.03 (1.36)	2.68 (1.49)	2.34 (1.47)	2.87 (1.41)	2.80**
Distance from the farm to the main road (minutes)	46.97 (22.38)	44.90 (21.99)	53.10 (28.99)	66.25 (23.00)	48.60 (24.26)	8.72*

Abbreviations: FS, Food Secure; MFI, Moderately Food Insecure; MFS, Marginally Food Secure; S.D., Standard deviation; SFI, Severely Food Insecure.

* *p* < 0.01.

** *p* < 0.05.

*Source:* Own survey (2024).

In Table 2, the following acronyms are used to indicate household food security categories: FS, Food Secure; MFS, Marginally Food Secure; MFI, Moderately Food Insecure; and SFI, Severely Food Insecure. Household head age varied across categories, with the oldest heads in food secure households (FS: 50.07 ± 8.17; MFS: 47.65 ± 9.25) and the youngest in food insecure households (MFI: 45.22 ± 7.54; SFI: 44.90 ± 8.49; *F* = 6.19, *p* < 0.05). This finding likely reflects the role of experience in enhancing food security, suggesting that younger household heads could benefit from targeted capacity‐building. Educational attainment followed a similar pattern, with FS and MFS heads being more educated (6.07 ± 2.87 and 6.39 ± 2.68 years) than MFI and SFI heads were (4.92 ± 2.38 and 5.53 ± 3.04 years; *F* = 5.82, *p* < 0.05), highlighting the importance of knowledge in agricultural management and income generation, and suggesting that adult education programs are potential interventions. Household composition showed a gradient: the proportion of children under 14 years of age increased from FS (0.48 ± 0.14) and MFS (0.47 ± 0.15) to MFI (0.65 ± 0.11) and SFI (0.67 ± 0.07; *F* = 49.79, *p* < 0.01), indicating that higher dependency ratios consistently increase vulnerability. Agricultural resources also differed: FS and MFS households held more land (1.18 ± 0.90 and 1.07 ± 0.78 ha) and cultivated more crops (2.88 ± 1.37 and 3.03 ± 1.36) than MFI and SFI households did (land: 0.88 ± 0.47 and 0.79 ± 0.47 ha; crops: 2.68 ± 1.49 and 2.34 ± 1.47; farmland: *F* = 3.90, *p* < 0.05; crops: *F* = 2.80, *p* < 0.05), suggesting that larger landholdings and cultivating more crops may enhance household resilience. The distance to the main road was greater for SFI households (66.25 ± 23.00 min) and MFI (53.10 ± 28.99) than for FS households (46.97 ± 22.38 min; *F* = 8.72, *p* < 0.05), underscoring the role of connectivity in accessing markets and services.

Post hoc Tukey HSD comparisons of continuous covariates across food security categories (FS, MFS, MFI, and SFI) revealed several significant differences. Compared with MFI and SFI households, FS households had older heads and higher educational attainment. Compared with MFI and SFI households, FS and MFS households had fewer children under 14, whereas FS households had larger farmland than MFI and SFI households did. The distance from the farm to the main road was greatest for SFI households, highlighting geographic isolation as a risk factor. The number of crops grown did not differ significantly across the four food security categories, suggesting that merely cultivating more crops may not directly improve household food security in this context. Overall, these results emphasize the need for integrated interventions targeting human capital, agricultural resources, dependency management, and rural infrastructure.

Table 3 shows the associations between categorical predictors and household food security status in Ada'a District, with food security category acronyms as defined in Table 2. Compared with female‐headed households (FS: 6.4%; MFS: 11.8%), male‐headed households (FS: 20.5%; MFS: 35.1%) (*χ* ^2^ = 11.21, *p* = 0.011) were more likely to be food secure. This finding aligns with prior evidence that female‐headed households face cultural constraints, heavy time burdens, limited access to appropriate technologies, and restricted mobility, which limit livelihood participation and weaken food security (Mekonen et al. 2023; Aweke et al. 2022), whereas male‐headed households enjoy better access to diverse food sources and sociocultural advantages that support wider dietary options and greater food security (Mohammed et al. 2025; Beyene et al. 2024). Similarly, married‐headed households were predominantly food secure (FS: 26.4%; MFS: 44.3%), whereas single‐headed households were concentrated in the moderately (MFI: 10.8%) and severely food insecure categories (SFI: 3.8%; *χ* ^2^ = 152.21, *p* < 0.001). Our findings align with prior evidence that marital status enhances the pooling of available resources, the economic scale of consumption items purchased, and household labor allocation and coping capacity (Cahyono and Tokuda 2024; Mekonen et al. 2023; Mohammed et al. 2025).

**TABLE 3:** Association between categorical predictors and household food security status, Ada'a District (*n* = 424).

Variable	Category	CARI ‐FSI				Overall percent	Chi‐square
		FS	MFS	MFI	SFI		
Sex of household head	Male	20.5	35.1	16.5	7.1	79.2	11.21**
	Female	6.4	11.8	2.1	0.5	20.8	
Marital status of household head	Single	0.5	2.6	10.8	3.8	17.7	152.21***
	Married	26.4	44.3	7.8	3.8	82.7	
At least one adult male present (≥ 18 years)	Yes	14.9	24.5	11.1	3.8	54.3	1.47^NS^
	No	12.0	22.4	7.5	3.8	45.7	
Seasonal labor migration	At least one member migrated	23.6	42.7	7.5	3.1	76.9	112.20***
	Otherwise	3.3	4.2	11.1	4.5	23.1	
Access to credit	Yes	17.2	30.0	8.0	4.7	59.9	11.53***
	No	9.7	17.0	10.6	2.8	40.1	
Access to agricultural extension service	Yes	19.3	35.1	18.6	7.5	80.7	36.46***
	No	7.5	11.8	—	—	19.3	
Access to irrigation	Yes	7.5	14.9	16.3	6.4	45.0	104.77***
	No	19.3	32.1	2.4	1.2	55.0	
Household head's perceived rainfall variability	Perceived variability	25.2	43.6	7.3	2.1	78.3	159.79***
	No perceived change	1.7	3.3	11.3	5.4	21.7	
Household head participates in at least one CBO	Yes	8.0	11.1	14.9	6.6	40.6	108.70***
	No	18.9	35.8	3.8	0.9	54.4	
Pest and disease infestation	Occurred	4.7	6.6	2.4	1.7	15.3	2.16^NS^
	Not occurred	22.2	40.3	16.3	5.9	84.7	
Adoption of high yield varieties	Adopter	10.1	17.7	18.6	7.5	54.0	128.04***
	Nonadopter	16.7	29.2	—	—	46.0	

Abbreviations: FS, food secure; MFI, moderately food insecure; MFS, marginally food secure; NS, not significant; SFI, severely food insecure.

** *p* < 0.05.

*** *p* < 0.01.

*Source:* Authors' survey results (2024).

The presence of at least one adult male (≥ 18 years) in the household did not significantly influence any of the four food security categories (*χ* ^2^ = 1.47, *p* = 0.689), suggesting that male adults alone do not confer protection in our context. Rather, household food security appears to depend on the overall capacities of all household members. In contrast, Mbhenyane and Tambe (2024) in South Africa reported that the total number of adults in a household (both males and females) was positively and significantly associated with food security, whereas the number of male household members alone did not have a significant influence.

Households with at least one member engaged in seasonal migration were more concentrated in the food secure (FS: 23.6%) and marginally food secure (MFS: 42.7%) categories, with fewer households in the moderately (MFI: 7.5%) and severely food insecure (SFI: 3.1%) categories (*χ* ^2^ = 112.20, *p* < 0.001). At the descriptive level, this pattern may suggest that seasonal migration provides supplementary income or food access, resulting in improved food security. However, evidence from drought‐prone Northeast Ethiopia shows that seasonal migration is often driven by climatic and economic shocks, and many migrant households remain food insecure because of reduced on‐farm labor and constrained local food availability (Asefawu 2022). Similarly, conflict‐induced migration in Sudan has been associated with increased levels of food insecurity (Abushama et al. 2024). Because the bivariate association does not account for confounding factors such as household wealth, labor allocation, and farm characteristics, the econometric results revealed that, once these factors are controlled for, seasonal migration functions primarily as a distress coping strategy that diverts labor from productive activities and insufficiently offsets production losses, thereby increasing the risk of moderate or severe food insecurity (see Section 4.3).

Access to credit and agricultural extension services also varied significantly across food security categories. Households with access to credit were predominantly food secure (FS: 17.2%) or marginally food secure (MFS: 30.0%), compared with households without credit access (FS: 9.7%; MFS: 17.0%; *χ* ^2^ = 11.53, *p* < 0.01). This pattern likely reflects the role of credit in enabling consumption smoothing, productive investment, and coping with shocks (Ahmed and Ambinakudige 2025; Boltana et al. 2023). Similarly, access to agricultural extension services was more common among food‐secure households with extension access (FS: 19.3%, MFS: 35.1%) than among food‐secure households without extension access (FS: 7.5%, MFS: 11.8%; *χ* ^2^ = 36.46, *p* < 0.001). Overall, 54.4% of combined households with access to extension services were food secure (FS + MFS), whereas only 19.3% of combined food secure households without access to extension services were food secure. This finding underscores the role of agricultural extension in improving access to information, strengthening farming practices, and enhancing food security. This finding is consistent with that of Aremu et al. (2025), who reported that agricultural extension and advisory services significantly improve household welfare and food security outcomes in Ghana. This finding aligns with that of Mugizi (2025), who reported that households receiving extension advice for crop production are more likely to adopt improved seeds, organic and inorganic fertilizers, irrigation, and conservation farming practices, mechanisms that are strongly associated with higher agricultural productivity and improved food security.

The adoption of high‐yield varieties was highest among FS and MFS households (10.1% and 17.7%, respectively) and lower among MFI and SFI households (18.6% and 7.5%; *χ* ^2^ = 128.04, *p* < 0.001). This finding aligns with recent studies showing that, compared with nonadopters, adopters of improved seed varieties achieved greater production and greater consumption, both in terms of calorie intake and food and nonfood expenditures, thereby directly improving their food security status (Fayera 2024; Tesfaye 2025). Households' perceptions of rainfall variability were strongly associated with food security status. Food secure (25.2%) and moderately food secure (43.5%) households were more likely to perceive rainfall changes, whereas perceptions were lower among moderately food insecure (11.3%) and severely food insecure (5.4%) households (*χ* ^2^ = 159.79, *p* < 0.001), suggesting that climate awareness may reflect households' adaptive capacity and inform targeted interventions. These findings align with evidence from the Horn of Africa (Bitew and Minale 2025; Omer et al. 2025; Aniye et al. 2025), which shows that farmers' climate perceptions closely match observed climate trends and can be leveraged to design strategies for mitigating climate‐related food insecurity.

Participation in community‐based organizations (CBOs) was greater among moderately (MFI: 14.9%) and severely food‐insecure (SFI: 6.6%) households than among food‐secure (FS: 8.0%) and moderately food‐secure (MFS: 11.1%) households (*χ* ^2^ = 108.70, *p* < 0.001), suggesting that more vulnerable households may engage with CBOs as a coping strategy. These raw cross‐tab observations, which do not account for other household factors, contrast with findings from Mozambique (Tadesse et al. 2025) and Latin America (Monteza‐Quiroz et al. 2025), where participation in community‐based financial groups or broader social capital was associated with improved household food security. Similarly, Yusriadi (2025) reported that strong social capital through agroforestry networks supports sustainable food security. The divergence in our context may indicate that CBO participation may be largely reactive, with food‐insecure households joining to seek support rather than immediately improving food security, highlighting the importance of CBO type, resources, and effectiveness in mediating household outcomes. Pest and disease occurrence was not significantly associated with any food security category (*χ* ^2^ = 2.16, NS), possibly because households were able to manage outbreaks through local knowledge or timely interventions, or because the effects of pests and diseases were relatively uniform across the district, limiting variation in food security outcomes, or because of differences in household resilience.

Overall, the findings suggest that FS and MFS households consistently have advantages in headship, marital status, migration, credit, extension, adoption of improved varieties, and climate awareness, whereas MFI and SFI households show heightened vulnerability. These findings underscore the need for tailored interventions addressing the specific vulnerabilities of each food security category, particularly single‐ and female‐headed households, less educated heads, and those lacking access to resources and market connectivity.

### 4.2 Analysis of Food Security Indicators

Table 4 illustrates that households may appear food secure on one indicator but not on others, highlighting the versatility of the CARI model.

**TABLE 4:** Construction of the composite CARI‐FSI and its indicators, Ada'a District (*n* = 424).

Domain		Indicator	Food Secure (1)	Marginally Food Secure (2)	Moderately Food Insecure (3)	Severely Food Insecure (4)
Current Status	Food consumption	FCS	66.0% Acceptable*	—	24.8% Borderline*	9.2% Poor*
Coping capacity	Economic vulnerability	FES	51.4% < 50%*	20.3% 50%–65%*	10.8% 65%–75%*	17.5% > 75%*
	Asset depletion	LCS‐FS	70.3% Not adopted*	11.1% Stress*	7.8% Crisis*	10.8% Emergencies*
CARI—Food Security Index (FSI)			26.9% (*n* = 114)	46.9% (*n* = 199)	18.6% (*n* = 79)	7.5% (*n* = 32)

*Note:* *Thresholds, table color, and design adapted from WFP (2015).

*Source:* Own survey result (2024).

The CARI‐FSI classified 26.9% of households as food secure, 46.9% as marginally food secure, 18.6% as moderately food insecure, and 7.5% as severely food insecure, indicating that over 25% of households remain food insecure despite Ada'a District's reputation as a food surplus area. While 66% of the households in the study area consumed acceptable food groups, their diets were overwhelmingly dominated by cereals (99.8%), with limited consumption of pulses, dairy, and other animal‐source foods (Shumiye et al. 2025), highlighting significant nutritional gaps. Combined with financial strain for some households (20.3% spent 50%–65% of income on food, 17.5% spent > 75%), these findings underscore persistent vulnerability and the need for targeted, multifaceted interventions to strengthen resilience.

### 4.3 Food Security Determinants Among Rural Households in Ada'a District

Predictor variables were selected on the basis of a review of the literature and their relevance for the multivariate analysis of food security in the context of this study. Six continuous and eleven categorical variables were retained in the ordinal logistic regression model (Table 5).

**TABLE 5:** Ordered logistic regression results (*n* = 424).

Explanatory variables	Estimate	Std. error	Wald	Sig.	Odds ratio (OR)
Age of household head in years	−0.031	0.012	6.935	0.008***	0.97
Proportion of children aged 14 and under	1.937	0.771	6.315	0.012**	6.94
Marital status of household head (1 = Single)	1.525	0.313	23.775	0.000***	4.60
Sex of household head (1 = Male)	−0.273	0.251	1.177	0.278^NS^	0.76
*At least one adult male household member* aged ≥ 18 years present (1 = Yes)	0.299	0.211	2.016	0.156^NS^	1.35
Education level of household head in years of schooling	−0.024	0.037	0.410	0.522^NS^	0.98
Farmland size in hectare	−0.330	0.140	5.542	0.019**	0.72
Number of crops grown	−0.020	0.075	0.070	0.791^NS^	0.98
Seasonal labor migration (1 = At least one household member migrated for seasonal work)	0.977	0.268	13.276	0.000***	2.66
Participation in community‐based organizations (CBOs CBO refers to Iqub and Iddir. Iqub is a rotating savings scheme with pooled cash distributed among members, while Iddir is a community association that collects contributions to cover burial and related expenses.) (1 = Household head participated in at least one CBO)	−0.599	0.234	6.563	0.010**	0.55
Access to extension service (1 = Yes)	−0.737	0.277	7.095	0.008***	0.48
Access to credit (1 = Yes)	−0.062	0.212	0.085	0.771^NS^	0.94
Adoption of high yield varieties (1 = Adopter)	−0.971	0.235	17.128	0.000***	0.38
Access to irrigation (1 = Yes)	−0.637	0.233	7.479	0.006***	0.53
Distance from farm to main road (minutes)	0.011	0.004	6.695	0.010**	1.01
Perceived rainfall variability (1 = Perceived rainfall variability)	1.293	0.300	18.603	0.000***	3.64
Pest and disease infestation (1 = Occurred)	0.466	0.281	2.756	0.097*	1.59

Abbreviation: NS, not significant.

* *p* < 0.10.

** *p* < 0.05.

*** *p* < 0.01.

*Source:* Own survey result (2024).

Model diagnostics confirmed the robustness of the results. The proportional odds assumption, tested via a chi‐square statistic (*χ* ^2^ = 13.343, *p* = 0.999), was met, indicating consistent effects of predictors across all categories. Multicollinearity was assessed via Pearson correlations and variance inflation factors (VIFs), which ranged from zero to 0.37 (in absolute value) and from 1.022 to 1.470, respectively. Both measures are well below conventional thresholds (*r* > 0.7; VIF > 5), indicating that there is no concern and supporting the reliability of the regression estimates. Annex D in Data S1 reports the VIF values and Spearman correlation matrix. The analysis of model fit shows a significant improvement over the intercept‐only model, with a chi‐square value of 307.735 and a *p* value of 0.000, indicating that the seventeen predictors are jointly statistically significant. The Pearson goodness‐of‐fit tests yield *p*‐values above 5%, confirming good model fit. The Nagelkerke pseudo‐*R* ^2^ of 0.566 shows that the seventeen predictors improved food security prediction by 56.6%, with the rest explained by other factors. Table 5 presents the ordered logistic regression results.

The estimated effect of the household head's age on the outcome variable was negative and statistically significant (*β* = −0.031, *p* = 0.008). Since the household food security variable is coded from 1 = food secure to 4 = severely food insecure, the negative coefficient indicates that households headed by older individuals are more likely to be in the food secure or marginally food secure categories. The odds ratio (0.97) implies that each additional year of age increases the likelihood of being in a lower food insecurity category (1 = food secure or 2 = marginally food secure) by approximately 3%, with other factors held constant. This finding implies that accumulated farming experience, social capital, and risk‐management capacity may help offset age‐related constraints in the study context. Several studies suggest that household experience and tenure enhance resilience to food shocks, with older household heads generally experiencing lower levels of food insecurity (Aweke et al. 2022; Awoyemi et al. 2023; Mekonen et al. 2023; Mohammed et al. 2025; Oyato et al. 2024). For example, in northwestern Ethiopia, the experience and social or economic capital of older household heads were found to improve household outcomes (Nega et al. 2025). However, evidence from Sub‐Saharan Africa highlights the mixed nature of these effects, as older households in some contexts still face high levels of food insecurity, with severe and moderate insecurity reported in 6%–87% and 8%–48% of households, respectively (Saha et al. 2021). Similarly, studies in Nigeria have shown that older households tend to have lower dietary diversity (Amao et al. 2023) and are more vulnerable to food insecurity when labor availability, assets, or technology adoption are limited (Tefera et al. 2022). These findings suggest that the influence of age on food security is context dependent, likely reflecting a balance between accumulated experience, stable land access, intergenerational labor support, and livelihood diversification, and underscore the need to disentangle age effects from life cycle, asset, and institutional factors when designing food security interventions.

The positive and statistically significant effect of the proportion of children aged 14 years and under (*β* = 1.937, OR = 6.94, *p* = 0.012) indicates that an increase in the share of young dependents is associated with nearly sevenfold greater odds of a household being moderately or severely food insecure, with all other factors held constant. This finding underscores the central role of the dependency burden, rather than household size per se, in shaping food security risk. As the ratio of nonworking children to productive adults increases, household consumption needs rise faster than income‐generating capacity does, intensifying vulnerability. In other words, fertility and dependency ratios, rather than absolute household size, are key pathways through which food access is constrained. Our results corroborate empirical studies showing that households with more dependents than working adults have higher odds of food insecurity, confirming that household demographic structure significantly affects food access (Argaw 2019; Jambo and Derso 2025; Mamo et al. 2024; Sisha 2020). Collectively, these findings highlight the importance of designing food security interventions that explicitly account for household age composition, particularly in contexts where social protection and child‐focused nutrition programs are limited.

Marital status had a strong positive and statistically significant effect (*β* = 1.525, OR = 4.6, *p* < 0.01), indicating that single household heads (i.e., unmarried, widowed, or separated) were associated with nearly five times greater odds of being moderately or severely food insecure than were married households. This pronounced effect underscores the protective role of marriage in pooling labor, income, and decision‐making capacity, as well as in strengthening access to social and informal support networks. More importantly, the findings highlight that single‐headed households, predominantly females because men are more likely to remarry after divorce or widowhood, remain structurally disadvantaged, suggesting that greater food insecurity reflects systemic and cultural constraints rather than individual characteristics alone. Our findings align with prior evidence that joint household management enhances resilience to food shocks (Chege et al. 2016; Guyalo 2025; Yusuf et al. 2015) and that married household heads are less likely to fall into poor household food consumption categories (Awoyemi et al. 2023) or to have unmet healthcare needs (Odunyemi et al. 2024).

The sex of the household head had a negative but statistically insignificant coefficient (*β* = −0.273, *p* = 0.278), suggesting that male‐headed households tend to be more likely to be food secure or marginally food secure than female‐headed households are, although this association is not statistically significant. While the direction of the effect is consistent with studies linking male headships to better access to productive resources (Guyalo 2025; Hailu 2023; Mohammed et al. 2025), the lack of statistical significance potentially indicates that headship sex alone is an insufficient predictor of food security in this context. To probe this further, we examined whether the presence of at least one adult male household member (aged ≥ 18 years) was associated with household food security status. The estimated coefficient was positive but statistically insignificant (*β* = 0.299, *p* = 0.156), indicating a tendency toward a greater likelihood of being moderately or severely food insecure among households with an adult male present; however, this association is not statistically significant. Taken together, these results suggest that the increased vulnerability of single‐headed, predominantly female‐headed, households reflects structural disadvantages in access to land, credit, labor markets, and social networks, rather than household composition or biological sex, a finding that is consistent with Fikre and Tsige (2025), who reported similar patterns among “*Enset*” (false banana) growers in southern Ethiopia. These findings highlight marital status and gendered power relations, rather than sex or adult presence per se, as the more decisive determinants of household food security.

Household head education had a negative but statistically insignificant effect (*β* = 0.04, *p* = 0.522), suggesting that although higher education may be associated with a greater likelihood of being food secure or marginally food secure, its influence in this context is likely overshadowed by more immediate factors such as access to resources, household income, and exposure to shocks. In contrast, studies in Ethiopia report that higher household head education is significantly associated with lower food insecurity, whereas low education corresponds to higher food insecurity incidence (Seyoum et al. 2025), and other studies confirm that low education often coexists with additional household‐level food insecurity risk factors (Bogale et al. 2025). Some studies have shown mixed results: higher education is generally linked to greater income, increased participation in farm and nonfarm activities, and the adoption of sustainable farming practices, which can enhance household food resilience (Asante et al. 2024; Borku et al. 2024; Douyon et al. 2022; Hodjo et al. 2024). However, in some contexts, educated household heads experience similar or even greater food insecurity than less educated heads do (Aboye et al. 2024; Spieker et al. 2022), reflecting the influence of local labor markets, livelihood options, and structural constraints. Collectively, these findings indicate that the effect of education on household food security is context dependent, and is mediated by socioeconomic conditions and structural factors, underscoring the need to interpret education's role within specific local contexts when interventions are designed.

Farmland size had a negative and statistically significant effect (*β* = −0.333, OR = 0.72, *p* < 0.05), indicating that households with larger landholdings are more likely to be food secure or marginally food secure. Holding other factors constant, each additional hectare increases the odds of being in a food secure or marginally food secure category by approximately 28%. These results underscore land access as a key determinant of household resilience, as it supports both agricultural production and income diversification. Our findings align with those of prior studies (Dosso et al. 2024; Gebrehiwot et al. 2024; Guyalo 2025; Seyoum et al. 2025), which reported that each additional hectare increases the likelihood that a household is food secure. Land access also influences farmers' decisions regarding sustainable agricultural practices and resource management. Consistent with classic microeconomic theories of production and household optimization, greater endowments of productive assets enable households to allocate resources more efficiently, diversify outputs, and smooth consumption, thereby contributing to improved food security outcomes. In contrast, some studies suggest that larger farmlands do not necessarily guarantee food security. For example, Aweke et al. (2022) reported that larger landowners may face financial and technical constraints that limit the adoption of modern inputs, leading to extensification rather than intensification, which can reduce the food security benefits of additional land.

The number of crops grown had a negative but statistically insignificant effect (*β* = −0.02, *p* = 0.838), suggesting that cultivating multiple crops alone is not associated with a greater likelihood of a household being food secure or marginally food secure in this context. This finding may reflect constraints such as limited market access or low‐value crops, which prevent households from translating production into sufficient food or income. Similarly, Odhiambo et al. (2024), in a study on food crop diversification in Busia County, Kenya, reported a very weak and statistically insignificant relationship between crop diversification and household food security, showing that simply growing a wider range of crops does not automatically improve food security outcomes. In contrast, a recent study by John (2024), which used panel data from Tanzania, revealed that indigenous and exotic crop diversity significantly improved household food consumption, a key dimension of food security. Similarly, Sawadogo and Sawadogo (2025) reported that greater agricultural diversification in Burkina Faso increased the likelihood of improved household food security, largely through its positive effect on household income. Consistent with household production theory, asset endowments enhance well‐being only when they can be effectively converted into consumption or utility, suggesting that crop quantity alone is insufficient without complementary resources and market opportunities. Moreover, growing a greater number of crops is likely to have a positive effect on household food security when paired with supportive interventions and appropriate varietal choices.

Households with at least one member who migrated for seasonal work had a positive and statistically significant effect (*β* = 0.977, OR = 2.66, *p* < 0.01), suggesting that, holding other factors constant, these households are approximately three times more likely to fall into moderate or severe food insecurity categories than households without seasonal migrants. This finding indicates that seasonal migration may function primarily as a distress coping strategy driven by unstable income or limited local employment, which can reduce farm labor and lower productivity. From a household production and labor allocation perspective, diverting labor away from farm activities does not necessarily increase utility if the income earned is insufficient to offset the associated losses. Our findings align with those of prior studies showing that seasonal migration in drought‐prone northeast Ethiopia reduces household food availability and access, leaving many migrant households food insecure (Asefawu 2022), whereas conflict‐induced migration in Sudan increases moderate to severe food insecurity among rural and urban households (Abushama et al. 2024). In contrast, household head migration in northern Ethiopia can improve food security, with households experiencing migration having 2.61 times higher odds of being food secure than those without migration (Gebrehiwot et al. 2024). These studies collectively highlight that the impact of migration on household food security is context‐dependent: it may exacerbate vulnerability under stressful or forced conditions but improve outcomes when migration provides access to income or resources.

Participation in at least one community‐based organization (CBO) had a negative and statistically significant effect (*β* = −0.599, OR = 0.55, *p* < 0.05), indicating that, holding other factors constant, households engaged in CBOs have 45% greater odds of being in the food secure or marginally food secure categories. Our findings align with recent evidence showing that social capital and community engagement enhance household resilience by facilitating access to resources, collective support, and risk management. Consistent with social capital theory, such networks strengthen households' capacity to manage risks and buffer against shocks, thereby improving food security outcomes. For example, Monteza‐Quiroz et al. (2025) reported that greater social capital substantially reduces the prevalence of food insecurity by an estimated 23–25 percentage points, demonstrating the protective role of community networks in improving food access and stability. Similarly, Tadesse et al. (2025) reported that participation in Village Savings and Loan Groups in Mozambique significantly improved household food availability and reduced hunger by increasing asset ownership. Yusriadi (2025) further highlighted that active engagement in community networks enhanced food security outcomes in rural Indonesian communities, underscoring the role of social capital in mitigating vulnerability across diverse settings. In summary, these findings suggest that community participation and social networks constitute a critical pathway for strengthening household food security, complementing economic and productive resources.

Access to extension services had a negative and statistically significant effect (*β* = −0.737, OR = 0.48, *p* < 0.01), indicating that, holding other factors constant, households receiving extension support have 53% greater odds of being food secure or marginally food secure than those without access. This finding aligns with economic theory, which posits that well‐targeted extension services enhance knowledge, facilitate the adoption of improved technologies, and increase productivity, thereby strengthening food availability and stability at the household level. Similar evidence from Tanzania showed that extension advice for crop production increased agricultural productivity and subsequently improved household food security by promoting the adoption of better farming practices (Mugizi 2025). Similarly, a nationally representative panel study in Ghana revealed that access to agricultural extension services significantly increased household food consumption and dietary diversity, reinforcing the role of extension in reducing food insecurity (Aremu et al. 2025).

Access to credit had a negative effect, but the effect was not statistically significant, implying that credit alone may not be sufficient to increase the likelihood of households achieving food security or marginal food security in this setting. Constraints such as high interest rates, limited affordability, poor accessibility for vulnerable households, and inadequate targeting may limit the effectiveness of credit services, whereas household capacity to use loans productively also affects outcomes. Consistent with our findings, Ahmed and Ambinakudige (2025) reported in northern Bangladesh that restricted loan access and high interest rates constrained farmers' productive potential, with nearly half of respondents reporting that loan repayments negatively affected food consumption, particularly after natural disasters. Conversely, Jambo and Derso (2025) reported a positive association between credit access and household food security in the East Bale Zone of Ethiopia, indicating that when coupled with enabling conditions, credit can improve food availability and overall food security. Collectively, these findings highlight the context‐dependent nature of credit's impact, showing that while it can support food security, its effectiveness depends on affordability, accessibility, and households' capacity to utilize loans productively.

The adoption of high‐yield varieties (HYVs) (*β* = −0.971, OR = 0.38, *p* < 0.01) and access to irrigation (*β* = −0.637, OR = 0.53, *p* < 0.01) significantly increased the likelihood of households being food secure or marginally food secure. Specifically, households that adopted HYVs had approximately 62% higher odds, whereas those with irrigation access had approximately 47% higher odds of achieving food security or marginal food security. These substantial gains are consistent with recent evidence indicating that improved agricultural technologies increase productivity, stabilize food availability, and reduce vulnerability to climatic and market shocks. Consequently, scaling up access to HYVs and irrigation, particularly for resource‐poor and high‐risk households, represents a key strategy for strengthening rural food security and resilience. Similar findings have been reported in the North Wollo Zone, Ethiopia, where the combined adoption of climate‐smart practices, including HYVs and irrigation, led to significant increases in food variety and marked reductions in household food insecurity, confirming that integrated technology use strengthens food availability and resilience (Tessera and Molla 2025). Likewise, studies on improved rice variety adoption in northwestern Ethiopia have shown that adopters produce and consume more food, translating into improved household food security outcomes (Tesfaye 2025). Evidence from small‐scale irrigation schemes in Ethiopia further indicates that irrigation access enhances agricultural productivity and household food security status among rural households (Abadi et al. 2025). Collectively, these findings underscore that promoting HYVs and irrigation, especially when combined with supportive practices, can effectively increase food security in smallholder contexts.

Distance from the farm to the main road (*β* = 0.011, OR = 1.01, *p* < 0.05) and the occurrence of pest and disease infestations (*β* = 0.466, OR = 1.59, *p* < 0.10) were associated with a greater likelihood of households being moderately or severely food insecure. The odds ratio of 1.01 for distance indicates that for each additional minute it takes to reach the main road, the odds of a household experiencing moderate or severe food insecurity increase by approximately 1%. Similarly, the odds ratio of 1.59 for pest and disease occurrence implies that households affected by pests or diseases are approximately 59% more likely to be moderately or severely food insecure than those not affected. These results highlight that poor market access and crop health challenges are key drivers of household vulnerability, underscoring the urgent need for improved rural infrastructure, enhanced market connectivity, and effective pest and disease management to bolster food security. Recent empirical evidence reinforces these findings: in northern Ethiopia, greater market access, measured by proximity and frequency of engagement, is associated with higher dietary quality and improved food security, suggesting that households farther from markets face greater vulnerability due to transport costs and limited access to diverse foods (Balcha et al. 2025). Similarly, Africa‐wide studies have shown that longer travel times to markets correlate with greater food insecurity, emphasizing the importance of infrastructure and connectivity for food access (Benassai‐Dalmau et al. 2025). In Nigeria, pests and diseases are major contributors to on‐farm losses, exacerbated by knowledge gaps, economic constraints, and weak institutional support, which together undermine both pre‐ and postharvest food availability and increase hunger risk (Abulude 2025).

Households' perceptions of rainfall variability were positively and significantly associated with moderate or severe food insecurity (*β* = 1.293, OR = 3.64, *p* < 0.01). Specifically, households that perceive rainfall as irregular are more than three times more likely to experience moderate or severe food insecurity. These results highlight the substantial influence of climate uncertainty on rural agricultural production and food security outcomes. Recent time‐series data from the Ethiopian Meteorological Institute (1990–2024) corroborate these household perceptions. Specifically, the coefficient of variation (CV) for rainfall remained consistently high over this period. Notably, volatility peaks occurred in 1995 (120%), 2009 (121%), and 2018 (167%), which collectively signify persistent atmospheric irregularity. The standardized anomaly index (SAI) further illustrates a history of frequent and severe droughts, particularly during 1990 (−1.776) and 2022 (−2.098). Even in 2024, a strong positive SAI (+1.840) suggested above‐average total rainfall. However, this volume coincided with a high CV of 77%. This juxtaposition demonstrates that high aggregate moisture does not preclude risks associated with uneven distribution, such as delayed onset or intense, short‐duration bursts. The convergence of household perceptions and empirical metrics confirms that variability in both volume and timing increases food insecurity risk. These results emphasize the critical need for climate‐smart agriculture, robust early warning systems, and adaptive water management strategies to mitigate rural vulnerability. The synthesis above highlights a critical “*perception‐reality*” bridge. In climate science, the ability of rural populations to accurately perceive variability is a prerequisite for successful adaptation. The data confirm that even “*wet*” years (high SAI) can be food insecure years if the distribution (CV) is erratic.

Our findings corroborate recent evidence from the Horn of Africa, showing that rural households can accurately perceive climate variability, often relying on traditional knowledge and local indicators such as seasonal patterns, animal behavior, and changes in soil and vegetation. For example, Bitew and Minale (2025) reported that farmers' perceptions of declining rainfall and rising temperatures in the Upper Blue Nile Basin correspond with observed climate trends and are associated with production risks that reduce household food security. Similarly, Omer et al. (2025) in Somaliland reported that smallholder farmers recognize rainfall declines and drought events, whereas Aniye et al. (2025) in southeastern Ethiopia reported that farmers' perceptions of rising temperatures, erratic rainfall, and increased drought frequency closely matched meteorological records, with gender, education, and agroecological context influencing these perceptions. Collectively, these findings suggest that farmers' climate perceptions are reliable and can inform the design of targeted, community‐based interventions to mitigate climate‐related food insecurity.

## 5 Household Coping Strategies

### 5.1 Consumption‐Based Coping Strategies

Table 6 summarizes the short‐term coping strategies (rCSI) adopted by households during the 7‐day recall period prior to the survey. The most common strategies were reducing the number of daily meals (67%), borrowing food or money (59%), reducing meal size (57%), prioritizing children so that adults eat less (51%), and consuming fewer preferred foods (48%). These patterns indicate a reliance on consumption‐based coping, with households primarily managing shortages by reducing dietary quality and quantity rather than seeking productive or adaptive solutions. The high prevalence of borrowing suggests that many households face liquidity constraints during food stress, whereas the frequent prioritization of children highlights intrahousehold nutritional trade‐offs.

**TABLE 6:** Prevalence of short‐term coping strategies among rural households in Ada'a District (*n* = 424).

Coping strategies	Proportion of coping strategies adopted by rural households in Ada'a District					
	Never	Once	Sometimes (2–3 times)	Often (4–5 times)	All the time (6–7 times)	Most adopted coping strategy
Eating less preferred or less expensive foods	51.65	27.12	18.40	2.83	0.00	48.35
Borrowing food	40.57	18.16	31.13	9.91	0.24	59.43
Reduce number of meals eaten a day	33.49	22.88	39.62	4.01	0.00	66.51
Reducing portion sizes	43.16	20.75	29.01	6.37	0.71	56.84
Prioritizing children over adults for food	49.29	19.81	22.41	7.08	1.42	50.71

*Source:* Own survey data (2024).

### 5.2 Livelihood‐Based Coping Strategies (Asset‐Based)

**FIGURE 5:**
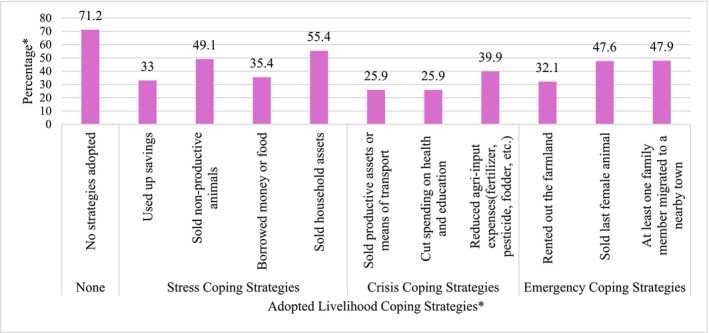
Adoption of livelihood coping strategies by rural households in Ada'a District. * Multiple responses allowed; totals within coping groups may exceed 100%. *Source:* Own survey data (2024).

The asset‐based coping activity index (LCS‐FS), which measures medium‐ and longer‐term coping capacity, has three distinct ordered levels. At each level, households reported undertaking different activities in the month and/or year prior to the survey date. The responses to food shortages among households in Ada'a District are illustrated in Figure 5.

As shown in Figure 5, the majority of households (71.2%) demonstrated some degree of resilience by not resorting to extreme coping mechanisms. Among the stress‐level strategies, over half (55.4%) sold household assets or goods, and nearly half (49.1%) sold nonproductive animals. At the crisis level, approximately 40% of respondents cut back on key farming inputs such as fertilizer and pesticides, whereas 26% reduced spending on health and education. Emergency responses were also significant; nearly 48% had at least one family member migrating to a nearby town, and a similar share (47.6%) sold their last female animal, which is critical for herd regeneration. These coping strategies, including selling productive assets, renting out farmland, or reducing farming investments, are concerning because they can undermine long‐term household resilience and exacerbate future food insecurity. This is especially concerning since the average size of farmland is only 1.05 ha, which is smaller than the national average of 1.10 ha (Ethiopian Statistical Service (ESS) and World Bank 2023). This limited landholding may constrain households' capacity to absorb shocks without resorting to asset depletion. Similar findings were reported by the same source at the national level, where rural households primarily depleted their savings or sold their livestock to cope with the top three shocks in order of impact: drought, an unusual increase in food prices, and an increase in input prices. This national pattern reinforces the local evidence from Ada'a District, illustrating a widespread vulnerability where systemic shocks directly trigger coping strategies that erode the very productive assets needed for recovery, thereby creating a cycle of diminishing resilience.

### 5.3 Determinants of Coping Strategy Choices Among Rural Households in Ada'a District

The consumption‐based coping frequency (CBCF) outcome variable in our sample of 424 households exhibited equidispersion, with a mean and variance of approximately 2.8, which is consistent with a Poisson model. However, a zero‐inflation problem was evident: 13% of households (*n* = 55) reported zero coping frequency, more than double the 6% expected under a standard Poisson distribution with *λ* = 2.8 (i.e., $P\left(Y=0\right)=e^{-2.8}\approx 0.06$). We therefore compared zero‐inflated Poisson (ZIP) and zero‐inflated negative binomial (ZINB) models. The overdispersion parameter alpha (*α*) in the ZINB model was negligible ($\alpha =1.6310^{-08}$), indicating significant overdispersion.

Furthermore, the ZIP model was preferred on the basis of model fit, yielding lower Akaike and Bayesian information criteria (AIC = 1149.436; BIC = 1189.93) than the ZINB model (AIC = 1151.436; BIC = 1195.983). Consequently, the ZIP model was selected as the best‐fitting specification. This model accurately captures the observed distribution by separately modeling the frequency of coping actions and the probability of being a “structural zero”, a household that does not engage in coping strategies. The results are presented in Table 7, which identifies statistically significant predictors in both the count (Poisson) and inflation (logit) components. These findings distinguish factors that influence the intensity of coping from those that predict the complete absence of coping behavior.

**TABLE 7:** Determinants of consumption‐based coping frequency, Ada'a District (*n* = 424).

Predictors	Coefficient	Robust Std. Err.	*z*	P > *z*	IRR^a^
**Count model (Poisson)**					
Perceived rainfall variability	0.092	0.008	11.410	0.000***	1.096
Market distance (km)	0.179	0.018	10.000	0.000***	1.196
Credit access (1 = yes)	−0.002	0.022	−0.080	0.935^NS^	0.998
Extension access (1 = yes)	−0.184	0.032	−5.820	0.000***	0.832
Market price shock (1 = yes)	0.204	0.038	5.340	0.000***	1.226
Remittance received (1 = yes)	−0.121	0.026	−4.730	0.000***	0.886
Constant	0.118	0.076	1.550	0.121	
**Inflate model (Logit)**					
Income (in Birr)	0.065	0.001	55.710	0.000***	
Farm size (in hectare)	0.592	0.223	2.650	0.008**	
Constant	−630.598	11.804	−53.420	0.000	

*Note:* **Significant at 5%; ***: significant at 1%.

Abbreviation: NS, non significant.

^*a*^ Incidence rate ratio.

*Source:* Own survey data (2024).

The Poisson component of the model (Table 7) revealed that market price shocks, market distance, and perceived rainfall variability were statistically significant (*p* < 0.01) predictors of higher coping frequency, with incidence rate ratios (IRRs) of 1.226, 1.196, and 1.096, respectively. Holding other variables constant, a one‐unit increase in each of these variables is associated with a 22.6%, 19.6%, and 9.6% increase, respectively, in the expected coping frequency. This finding indicates that households' reliance on consumption‐based coping strategies is significantly heightened by both climate‐related uncertainty and market‐related constraints. These findings are consistent with evidence that price shocks exacerbate rural household vulnerability under land constraints (Hodjo et al. 2024) and that limited market access is associated with poorer food security outcomes in rural and disadvantaged populations (Benassai‐Dalmau et al. 2025). Moreover, farmers' perceptions of erratic rainfall and drought appear to reflect actual climatic variability (Bitew and Minale 2025; Aniye et al. 2025; Omer et al. 2025). At the district level, our findings call for concrete, last‐mile interventions that strengthen market access and climate‐smart agricultural capacity. Local policies should prioritize (1) upgrading feeder roads to remote farming communities to reduce market travel time; (2) expanding access to affordable, climate‐resilient seeds and small‐scale irrigation; and (3) strengthening farmer cooperatives to increase collective bargaining power and cushion price shocks. Together, these measures directly target the key drivers of household coping behavior identified in this study.

Access to extension services and remittance receipts were significantly associated with lower CBCF (*p* < 0.01), with IRRs of 0.832 and 0.886, respectively. This translates to 16.8% and 11.4% reductions in the frequency of negative coping mechanisms, indicating a strong protective effect against food insecurity. Access to extension services likely enhances household resilience by improving agricultural knowledge, productivity, and adaptive capacity (*p* < 0.01), underscoring their importance for building human capital and reducing vulnerability in smallholder systems. This finding is consistent with evidence that, in Ghana, extension services increase agricultural productivity, reduce reliance on negative coping strategies (Aremu et al. 2025), and promote the adoption of home gardening techniques, thereby demonstrating the importance of access to knowledge and advisory services for reducing vulnerability (Asante et al. 2024). In Tanzania, extension advice better supports farm practices and adaptive capacity (Mugizi 2025), whereas in Ethiopia, extension services strengthen human capital and buffer households against production shocks (Baylie and Pércsi 2025).

Remittances (*β* = −0.121, IRR = 0.886, *p* < 0.01) were significantly associated with a lower frequency of coping strategies, indicating that they provided an alternative income source that smoothed consumption during periods of stress and acted as an informal safety net, helping households buffer against economic and climatic shocks. This finding is consistent with the literature on the role of external financial inflows and social capital in buffering households during shocks (Aniye et al. 2025; Asefawu 2022; Monteza‐Quiroz et al. 2025). Similarly, a study in Mozambique revealed that community‐based financial inclusion approaches can increase food availability through asset building, highlighting the potential of community‐driven financial strategies to improve the livelihoods of rural and low‐income women (Tadesse et al. 2025).

Access to credit (*β* = −0.002, IRR = 0.998, *p* > 0.10) was associated with only a 0.2% reduction in coping frequency and was statistically insignificant, indicating that the mere presence of credit programs is insufficient to meaningfully reduce households' reliance on negative coping strategies. Evidence from northern Bangladesh partially supports this interpretation. Although formal agricultural credit has expanded and reduced reliance on informal sources, nearly half of the farmers reported that loan repayments affect their food consumption and dietary choices, which highlights that the design of credit programs, including affordability, accessibility, and alignment with productive investments, is crucial for achieving meaningful food security benefits (Ahmed and Ambinakudige 2025). Other studies further emphasize that credit effectiveness depends on program design and linkages to productive use (Ahmed and Ambinakudige 2025), income‐smoothing opportunities (Tadesse et al. 2025), and the broader structural context (Awoyemi et al. 2023).

In the inflation (logit) component of the ZIP model (Table 7), which predicts the likelihood of a household reporting no coping strategies, both household income and farm size were positively and significantly associated (*p* < 0.01). Holding other factors constant, each additional Birr of income increased the log odds of reporting no coping by 0.065, and each additional hectare of farmland increased it by 0.596. These findings underscore the foundational role of economic capacity and productive assets in preventing the need for negative coping. Therefore, a dual‐track approach is recommended: first, supporting land access and sustainable intensification (e.g., through improved seeds, microirrigation, and soil conservation); second, creating pathways to diversify income (e.g., through local job creation, rural entrepreneurship grants, or skills‐for‐remittance programs). These efforts must be coordinated to reinforce one another. Our findings are consistent with those of prior studies showing that higher income enhances a household's purchasing power and ability to smooth consumption, thereby reducing vulnerability to shocks (Awoyemi et al. 2023; Bogale et al. 2025; Jambo and Derso 2025). Similarly, a larger farm size provides production potential and asset‐based security, stabilizes food consumption and amplifies the benefits of productivity‐enhancing interventions such as irrigation and climate‐smart crop intensification (Aboye et al. 2024; Abadi et al. 2025; Seyoum et al. 2025; Tessera and Molla 2025). Together, these findings underscore that strengthening household resilience requires integrated strategies targeting both income generation and land‐based productive assets.

## 6 Conclusion and Recommendations

This study examined the factors influencing food security and coping strategies among 424 rural households in Ada'a District, Central Ethiopia, via a cross‐sectional quantitative design with ordered logit and zero‐inflated Poisson regression models. The findings show that food security is shaped by a complex interplay of demographic, socioeconomic, institutional, environmental, and infrastructural factors. The composite food security index (CARI) revealed that approximately one quarter of the households were food insecure, with nearly half marginally food secure. Ordered logit regression revealed that household composition, resource endowments, institutional support, and environmental conditions were key determinants. Older household heads and households with larger farmlands, membership in community organizations, access to improved seeds, extension services, and irrigation tended to increase the likelihood of being food secure or marginally food secure. The presence of at least one adult male household member was not statistically significant, suggesting that male labor availability alone does not systematically improve household food security once other demographic, economic, and institutional factors are controlled for. Conversely, households with more children under 14 years of age, single‐headed households, and those engaged in seasonal labor migration increased the likelihood of being moderately or severely food insecure. Environmental stressors, particularly perceived rainfall variability and pest outbreaks, and limited market access further exacerbate the likelihood of moderate or severe food insecurity.

The study's application of a zero‐inflated Poisson model provided a nuanced understanding of coping behavior, distinguishing between the frequency of coping and the likelihood of avoiding it altogether. Environmental shocks, market distance, and economic stress increased reliance on coping, whereas extension services, remittances, higher income, and larger farm sizes played protective roles. These findings align with food entitlement decline theory, emphasizing that food insecurity often stems from failures in access and entitlements rather than absolute food shortages. Methodologically, this study demonstrates the utility of the CARI as a multidimensional measure of food security and highlights the effectiveness of the zero‐inflated Poisson model in disentangling the drivers of coping behavior. From a policy perspective, it provides the critical insight that, in Ada'a District, the inability to cope is structurally determined by household assets and income buffers, whereas the intensity of coping is driven primarily by exposure to covariate shocks, a distinction essential for effective targeting.

On the basis of empirical evidence, a coherent package of district‐level policy actions is recommended. Given the paramount role of environmental shocks, the initial priority should focus on integrating climate adaptation with social protection while concurrently planning for longer‐term infrastructure and institutional development. The following interventions are mapped to the principal drivers identified in this study:

- Systematically integrate climate‐resilient farming with social protection by bundling climate‐smart agriculture packages, such as drought‐tolerant seeds, microirrigation kits, and soil conservation training, with targeted support for vulnerable households, including single‐headed households and those with high dependency ratios. This requires close coordination between district agricultural offices and social protection programs to ensure that input subsidies and conservation incentives reach those most at risk.
- Last‐mile infrastructure should be strengthened through the rehabilitation and construction of feeder roads to remote Kebeles, particularly those facing the longest travel times to major markets. Leveraging community labor alongside district infrastructure budgets can reduce transport costs and improve timely access to both inputs and output markets.
- Local institutions should be reinforced by establishing and capacitating farmer cooperatives and women's savings groups to support collective input purchases, marketing, and off‐farm enterprises such as poultry, beekeeping, and handicrafts. This can be operationalized through cooperative start‐up grants and business development training delivered by district trade and industry offices in coordination with existing extension systems.
- Resilience can be promoted through financial inclusion by partnering with financial institutions and telecom providers to lower transfer costs, expanding mobile banking services in rural Kebeles, and delivering financial literacy programs that encourage the productive use of remittance income. District administrations can facilitate this by supporting rural agent banking outlets.
- Early warning and adaptive extension systems should be enhanced through the development of district‐level agroclimatic advisory services that integrate local rainfall monitoring with tailored extension messages delivered via SMS or radio. This can be supported by equipping development agents with smartphones and basic weather monitoring tools to provide timely guidance for plant decisions and pest management.

This study identifies context‐specific determinants of food security and coping strategies in Ada'a District, Central Ethiopia, although its cross‐sectional design precludes causal inference and self‐reported data may be affected by recall or social desirability bias. While findings may not be generalizable to other agroecological zones, they highlight the interconnected roles of farm productivity, market access, institutional support, and household resilience. With respect to challenging assumptions of uniform resilience in seemingly food‐secure areas, the results provide an evidence‐based framework for targeted interventions. Breaking the cycle of vulnerability requires policies that strengthen household assets, buffer communities against climate and market shocks, and protect the most vulnerable community from coping strategies that erode long‐term resilience. Future research should employ longitudinal designs, explore gender‐specific dynamics, and evaluate the effectiveness of integrated interventions in comparable rural contexts.

## Author Contributions

**Alem Shumiye:** conceptualized the study, conducted the data collection and analysis, developed the methodology, wrote the manuscript, and served as the corresponding author. **Degefa Tolossa:** contributed to the study design, methodology, and supervision, reviewed the manuscript, and endorsed it for publication. **Solomon Tsehay:** contributed to the study design, methodology, and data analysis; supervised the work; reviewed the manuscript; and endorsed it for publication. All the authors have read and approved the final manuscript.

## Funding

The authors have nothing to report.

## Ethics Statement

All data collection followed strict ethical standards. Ethical approval was obtained from the IRB of the College of Development Studies, Addis Ababa University (Approval No. 064/03/2023, March 8, 2024). Informed consent was secured from all participants, ensuring anonymity, confidentiality, voluntary participation, and the right to withdraw at any time.

## Consent

All the authors have provided explicit consent for publication.

## Conflicts of Interest

The authors declare no conflicts of interest.

## Supporting information


**Data S1:** fsn371588‐sup‐0001‐DataS1.docx.

## Data Availability

The data supporting the findings of this study are included in the manuscript and are available from the corresponding author upon reasonable request.

## Endnotes

^1^
A Kebele is the lowest administrative unit in Ethiopia.
