# Supplementary material for: Food Security Determinants and Coping Strategies Among Rural Households in Ada'a District, Central Ethiopia
Source: Food Sci Nutr. 2026 Mar 9;14(3):e71588. doi: 10.1002/fsn3.71588 (PMC12970487; doi:10.1002/fsn3.71588)
Supplement: Supplementary file 1 — Data S1: fsn371588‐sup‐0001‐DataS1.docx. [file FSN3-14-e71588-s001.docx]

**Annex A: Comparative summary of selected empirical studies on household food security determinants**

| **Purpose of the study** | **Authors & year** | **Context/Country** | **Food security measurement** | **Analytical framework** | **Key findings** |
| --- | --- | --- | --- | --- | --- |
| Analyze the socio-economic determinants of food security | **Cahyono & Tokuda (2024)** | **East Kalimantan province, which is largely food secure (85%) but faces persistent challenges among agricultural workers and rural residents (Indonesia).** | **Food Insecurity Experience Scale (FIES)** | **Binary logit** | Education, family size, land ownership, marital status, employment, residence, and retirement security significantly influence food security, whereas age, gender, and home ownership have minimal impact |
| Analyze food security determinants and coping strategies | **Alemayehu & Tesfaye (2024)** | Central highlands of Ethiopia | **Household Food Balance Model (HFBM**) | **Binary logit** | Major determinants of food security were land size, family size, household head’s age and education, livestock, credit, and market access. |
| Examine socio-economic factors affecting anxiety, insufficient food quality, and intake. | Pakravan-Charvadeh et al. (2022) | Afghan refugees residing in urban areas of Tehran province (Iran) | HFIAS. Households are classified as food secure or food insecure (marginal, moderate, or severe). | Multivariable regression | Employment, income, market proximity, and personal savings reduce food insecurity, whereas length of residence, house type, and number of children increase it. |
| Identify determinants of food security | **Aweke et al. (2022)** | **Central and North Gondar Zone, Ethiopia** | HDDS | **Binary logit** | Access to training, sex, family size, number of oxen, off-farm activities, farmland size, age, tropical livestock units, livelihood diversification, and household on-farm income were key factors influencing food security. |
| Develop and test a composite food security index to examine livelihood-related drivers of food security. | Mutea et al. (2019) | North-Western Mount Kenya Region, Kenya | The sum of standardized HDDS, FCS, and MAHFP scores, minus standardized CSI and HFIAS scores. | Multiple linear regression | Ownership of hand tools, off-farm income, own-food consumption, agro-ecological zone, and farm income positively affected food security, while crop pest infestation had a negative effect. |
| **Determine the sociodemographic factors affecting food security** | Ruhyana et al. (2020) | **Seemingly food secure Sumedang Regency, West Java Province (Indonesia).** | Energy intake relative to recommended dietary allowance and share of income spent on food. | **Binary logit** | Education, age, smoking habits, household size, rural residence, and employment status significantly affect food security, while households with agricultural income are more food insecure. |

*Source: Authors’ compilation*

**Annex B: Operational definition of food security determinants in Ada’a district**

| **Variables** | **Variable definition** | **Expected sign** | **Rationale** |
| --- | --- | --- | --- |
| **Dependent variable** | **Dependent variable definition** |  |  |
| Food security status | 1=Food secure, 2=Marginally food secure, 3=Moderately food insecure, 4=Severely food insecure |  | Lower values (1 or 2) indicate better food security, whereas higher values (3 or 4) indicate greater food insecurity. A positive coefficient/sign indicates a higher likelihood of being moderately or severely food insecure, while a negative coefficient/sign indicates a higher likelihood of being food secure or marginally food secure. |
| **Independent variables** | **Independent variables definition** |  |  |
| **Demographic factors:** |  |  |  |
| Age of household head | Age of household head in years | *Positive or Negative* | The age of the household head may have a dual effect: experience may increase food security (likelihood of being food secure or marginally food secure) (negative), while age-related burdens may increase the likelihood of moderate or severe food insecurity (positive). |
| Sex of household head | 1= Male  0 = Female | *Negative* | Male-headed households often have better access to assets and income opportunities, indicating a higher likelihood of being food secure or marginally food secure. |
| Marital status of the household head | 1 = Single (includes unmarried, widowed, and separated)  0 = Married. | *Positive* | Single-headed households often face labor and support constraints, increasing the likelihood of moderate or severe food insecurity (positive). |
| Proportion of children aged 14 and under | Percentage of household members aged 14 or younger, used as a proxy for child dependency. | *Positive* | Higher child dependency increases the consumption burden and reduces household resilience, increasing the likelihood of moderate or severe food insecurity (positive). |
| **Adult male present** | 1=at least one adult male household member aged ≥18 years;  0 = otherwise | *Negative* | The presence of adult males can contribute labor for farming or off-farm activities, improve access to income and resources, and influence household decision-making, all of which can enhance food security, with a negative sign indicating a greater likelihood of being food secure or marginally food secure. |
| **Socioeconomic factors:** |  |  |  |
| Education level of household head | Years of schooling | *Negative* | Education improves income potential, awareness, and food-related decision-making, with a negative sign hypothesized to indicate a greater likelihood of being food secure or marginally food secure. |
| Farmland size | Farmland size operated by the household head, in hectares. | *Negative* | Larger farmland size boosts production and food availability, with a negative sign indicating a greater likelihood of being food secure or marginally food secure. |
| Number of crops grown | Number of crop types cultivated by the household in the year preceding the survey. | *Negative* | Growing more crops reduces risk and broadens dietary variety, with a negative sign indicating a greater likelihood of being food secure or marginally food secure. |
| Seasonal labor migration | 1 = Household has at least one member who migrates seasonally for work  0 = Otherwise | *Positive or Negative* | Seasonal labor migration can bring income (negative), increasing the likelihood of being food secure or marginally food secure, but may reduce farm labor availability (positive), increasing the likelihood of moderate or severe food insecurity. |
| **Institutional factors:** |  |  |  |
| Participation in community-based organization (CBO) | A proxy for social capital: 1 = Household head participates in at least one CBO (e.g., *Iqub*^[[1]](#footnote-1)^, *Iddir*^[[2]](#footnote-2)^).  0 = Otherwise. | *Negative* | Social networks support access to resources and crisis coping, with a negative coefficient indicating a greater likelihood of being food secure or marginally food secure. |
| Access to extension service | 1= Yes  0 = Otherwise | *Negative* | Access to extension services improves productivity and food access, with a negative sign indicating a greater likelihood of being food secure or marginally food secure. |
| Access to credit | 1= Yes  0 = Otherwise | *Negative* | Access to credit is expected to enable investment in food production and smooth consumption, with a negative sign hypothesized to indicate a greater likelihood of being food secure or marginally food secure. |
| Adoption of high yielding varieties | 1= Adopter  0= Nonadopter | *Negative* | Adoption of high-yield varieties boosts productivity and food security, with a negative sign indicating a greater likelihood of being food secure or marginally food secure. |
| **Infrastructural factors:** |  |  |  |
| Access to irrigation | 1=Yes  0=No | *Negative* | Irrigation reduces reliance on erratic rainfall and supports stable food production, with a negative sign indicating a greater likelihood of being food secure or marginally food secure. |
| Distance to main road | Distance from farm to main road, measured in walking minutes. | *Positive* | Greater distance limits access to markets and services, worsening food access, with a positive sign indicating a greater likelihood of moderate or severe food insecurity. |
| **Environmental factors:** |  |  |  |
| Pest and disease infestation | 1 = Occurred  0 = Not occurred. | *Positive* | Reduces crop yields and leads to food loss, with a positive coefficient indicating a greater likelihood of moderate or severe food insecurity. |
| Household head’s perceived rainfall variability | 1=Perceived rainfall variability  0= No perceived change | *Positive* | Perceived climate variability often leads to yield loss and food shortages, with a positive sign indicating a greater likelihood of moderate or severe food insecurity. |

*Source: Authors’ own construction, based review of the literature*

**Annex C: Outcome and predictor variables and their hypothesized relationships**

| Variable | Measurement | Expected effect on coping frequency | Rationale |
| --- | --- | --- | --- |
| **Outcome variable:** |  |  |  |
| Consumption-based coping frequency (CBCF) | Number of consumption-based coping strategies used, ranging from 0 (none) to 5 (high). | - | Captures the intensity of short-term coping responses to food insecurity. |
| **Predictor variables** |  |  |  |
| Market price shock | A household experienced significant price increase in food and nonfood items (past 12 months) (1 = Yes; 0=No) | Positive | Rising prices reduce purchasing power, making food and nonfood items unaffordable and forcing households to adopt coping strategies more frequently. |
| Perceived rainfall variability | Households reported irregular or unpredictable rainfall patterns in the past 12 months (1 = Yes; 0 = No) |  | Perceived rainfall variability may lead to reduced agricultural productivity, increasing food insecurity and prompting households to adopt coping strategies more frequently. |
| Remittance received | Household reported receiving remittances in the past 12 months (1 = Yes; 0 = No) | Negative | Remittances improve household income and liquidity, reducing the need to rely on consumption-based coping strategies |
| Extension access | Household reported access to agricultural extension services in the past 12 months (1 = Yes; 0 = No) | Negative | Access to extension services enhances knowledge and resilience, helping households adopt improved practices and reduce reliance on coping mechanisms. |
| Market distance | Distance (in kilometers) from household to the nearest market | Positive | Greater distance limits access to goods and income opportunities, increasing reliance on coping strategies. |
| Income (in Birr) | Total monthly household earnings from all sources excluding remittances. | Negative | Higher income reduces the need to employ coping strategies. |
| Credit access | Households have accessed credit or loans in the past 12 months (1 = Yes; 0 = No) | Negative | Access to credit provides financial resources that help with smooth consumption and reduce coping needs. |
| Farmland size | \|  \| \| --- \|   Total farmland cultivated (in hectares) | Negative | A productive asset and proxy for production potential, larger farmland increases the likelihood of higher food production and income, thereby reducing the need for coping. |

*Source: Author’s construction*

**Annex D. Multicollinearity diagnostics**

**D.1. Variance inflation factors (VIF) and tolerance)**

| Labels | Predictors | Collinearity Statistics | |
| --- | --- | --- | --- |
|  |  | Tolerance | VIF |
| X1 | Age of household head (years) | 0.933 | 1.072 |
| X2 | Highest educational status of household head (years) | 0.956 | 1.046 |
| X3 | Proportion of children aged 14 and under | 0.694 | 1.442 |
| X4 | Farm land size (in hectares) | 0.896 | 1.116 |
| X5 | Number of crops grown | 0.890 | 1.124 |
| X6 | Distance from farm to the main road (minutes) | 0.942 | 1.062 |
| X7 | Marital status of household head | 0.735 | 1.361 |
| X8 | Seasonal labor migration | 0.785 | 1.273 |
| X9 | Gender of household head | 0.955 | 1.047 |
| X10 | Access to agricultural extension service | 0.859 | 1.164 |
| X11 | Perceived rainfall variability | 0.680 | 1.470 |
| X12 | Access to credit | 0.899 | 1.113 |
| X13 | Pest & disease infestation | 0.978 | 1.022 |
| X14 | Adoption of high yield varieties (HYV) | 0.772 | 1.296 |
| X15 | Household head participates in at least on CBO (e.g., Iddir or Equb) | 0.761 | 1.313 |
| X16 | Access to irrigation | 0.752 | 1.330 |
| X17 | Adult male present | 0.890 | 1.124 |
|  | **Minimum** |  | **1.022** |
|  | **Maximum** |  | **1.470** |
|  | **Mean** |  | **1.198** |

*Sources: Own survey data, 2024*

**D.2. Spearman’s correlation matrix of predictors**

|  | X1 | X2 | X3 | X4 | X5 | X6 | X7 | X8 | X9 | X10 | X11 | X12 | X13 | X14 | X15 | X16 | X17 |
| --- | --- | --- | --- | --- | --- | --- | --- | --- | --- | --- | --- | --- | --- | --- | --- | --- | --- |
| X1 | 1 |  |  |  |  |  |  |  |  |  |  |  |  |  |  |  |  |
| X2 | 0.04 | 1 |  |  |  |  |  |  |  |  |  |  |  |  |  |  |  |
| X3 | -0.14 | -0.04 | 1 |  |  |  |  |  |  |  |  |  |  |  |  |  |  |
| X4 | -0.14 | 0.07 | -0.09 | 1 |  |  |  |  |  |  |  |  |  |  |  |  |  |
| X5 | -0.05 | 0.02 | -0.05 | 0.20 | 1 |  |  |  |  |  |  |  |  |  |  |  |  |
| X6 | -0.02 | 0.05 | 0.09 | 0.06 | -0.06 | 1 |  |  |  |  |  |  |  |  |  |  |  |
| X7 | 0.07 | 0.00 | -0.36 | -0.05 | 0.03 | -0.05 | 1 |  |  |  |  |  |  |  |  |  |  |
| X8 | 0.06 | 0.08 | -0.30 | 0.15 | 0.02 | 0.02 | 0.26 | 1 |  |  |  |  |  |  |  |  |  |
| X9 | -0.02 | 0.02 | 0.09 | -0.10 | -0.08 | 0.08 | -0.07 | -0.03 | 1 |  |  |  |  |  |  |  |  |
| X10 | -0.05 | -0.03 | 0.16 | 0.03 | 0.10 | 0.01 | -0.16 | -0.11 | -0.03 | 1 |  |  |  |  |  |  |  |
| X11 | 0.16 | 0.08 | -0.37 | 0.06 | 0.09 | -0.09 | 0.37 | 0.34 | -0.11 | -0.19 | 1 |  |  |  |  |  |  |
| X12 | 0.02 | 0.03 | -0.14 | 0.07 | 0.06 | 0.04 | 0.04 | 0.16 | -0.02 | -0.22 | 0.04 | 1 |  |  |  |  |  |
| X13 | -0.08 | 0.01 | 0.01 | -0.01 | -0.01 | 0.01 | -0.01 | -0.06 | -0.06 | 0.03 | -0.03 | 0.03 | 1 |  |  |  |  |
| X14 | -0.11 | -0.04 | 0.31 | 0.00 | -0.05 | 0.09 | -0.30 | -0.21 | 0.12 | 0.16 | -0.33 | -0.07 | 0.04 | 1 |  |  |  |
| X15 | -0.08 | -0.09 | 0.27 | -0.03 | -0.19 | 0.09 | -0.26 | -0.27 | 0.07 | 0.15 | -0.36 | -0.09 | 0.05 | 0.33 | 1 |  |  |
| X16 | -0.14 | -0.07 | 0.28 | -0.02 | -0.04 | 0.15 | -0.34 | -0.25 | 0.04 | 0.20 | -0.32 | 0.00 | 0.08 | 0.30 | 0.24 | 1 |  |
| X17 | 0.01 | -0.04 | 0.24 | 0.00 | -0.05 | 0.07 | 0.02 | 0.04 | 0.07 | 0.03 | -0.04 | 0.00 | -0.02 | 0.04 | 0.05 | 0.07 | 1 |

*Sources: Own survey data, 2024*

**Note: X₁–X₁₇ are defined as described above.**

1. *Iqub is a traditional rotating savings scheme where members regularly save and take turns receiving the pooled cash, backed by peer collateral.* [↑](#footnote-ref-1)
2. *Iddir is a traditional community association where members contribute money to support burial and related expenses.* [↑](#footnote-ref-2)
